# Parameter estimation in behavioral epidemic models with endogenous societal risk-response

**DOI:** 10.1371/journal.pcbi.1011992

**Published:** 2024-03-29

**Authors:** Ann Osi, Navid Ghaffarzadegan

**Affiliations:** Department of Industrial and Systems Engineering, Virginia Tech, Blacksburg, Virginia, United States of America; Fundação Getúlio Vargas: Fundacao Getulio Vargas, BRAZIL

## Abstract

Behavioral epidemic models incorporating endogenous societal risk**-**response, where changes in risk perceptions prompt adjustments in contact rates, are crucial for predicting pandemic trajectories. Accurate parameter estimation in these models is vital for validation and precise projections. However, few studies have examined the problem of identifiability in models where disease and behavior parameters must be jointly estimated. To address this gap, we conduct simulation experiments to assess the effect on parameter estimation accuracy of a) delayed risk response, b) neglecting behavioral response in model structure, and c) integrating disease and public behavior data. Our findings reveal systematic biases in estimating behavior parameters even with comprehensive and accurate disease data and a well-structured simulation model when data are limited to the first wave. This is due to the significant delay between evolving risks and societal reactions, corresponding to the duration of a pandemic wave. Moreover, we demonstrate that conventional SEIR models, which disregard behavioral changes, may fit well in the early stages of a pandemic but exhibit significant errors after the initial peak. Furthermore, early on, relatively small data samples of public behavior, such as mobility, can significantly improve estimation accuracy. However, the marginal benefits decline as the pandemic progresses. These results highlight the challenges associated with the joint estimation of disease and behavior parameters in a behavioral epidemic model.

## Author summary

Understanding how society’s evolving risk perceptions alter social interactions and disease spread is key to building models that reliably project pandemic trajectories. This research focuses on systematic estimation of parameters in such models where disease dynamics and behavioral responses are intertwined. We find that even with perfect data and models, estimates of behavioral parameters are biased early in the pandemic, and data for at least one full wave of the disease are needed to have reliable estimates. This is mainly related to the time delay between pandemic risks, perceived risks, and public response. Our findings also show that conventional models that ignore risk response dynamics may replicate data well early on but eventually fail to uncover future waves. Additionally, incorporating small amounts of public behavior data (such as data samples from public mobility patterns) for model calibration significantly improves model accuracy, especially early in a pandemic. Moreover, the marginal benefit of additional behavioral data diminishes as the pandemic progresses. By understanding the challenges of estimating key parameters, we can build more reliable models for informed decision making during public health emergencies.

## 1. Introduction

During the COVID-19 pandemic, peoples’ behavior and reaction to the state of the pandemic played an important role in altering health outcomes across nations and communities [1, 2]. As COVID-19 cases and death tolls grew, public risk perception triggered human responses at the individual and governmental levels. These responses, which included non-pharmaceutical interventions (NPIs), helped flatten the curve of the pandemic and decrease the death toll [3–5]. However, as the number of cases declined, risk perception and compliance with NPI measures decreased, leading to more cases and new waves of the disease [6–8]. It is argued that such intertwined behavior-disease dynamics continues as long as an infectious virus is considered a major life-threatening risk [9].

With the recognition of the importance of human response in the pandemic trajectory, epidemic modeling scholars suggested developing models that incorporate human behavior [10, 11]. Such epidemic models peaked in the aftermath of the 2009 UK A/H1N1 pandemic [12]. These models often extend Susceptible-Exposed-Infected-Removed (SEIR)-like structures [13], to include interdependencies between the state of the disease, risk perception or fear, and change in average contact rate (e.g., [14–16]). For example, a common approach is to formulate a risk-response mechanism where infectivity rate is an inverse function of recent death to represent human response to change in death rate [2].

Despite advancements in modeling techniques, and the availability of more granular data from sources such as mobile phones or surveys, the challenge of validation and parameter identifiability for coupled behavior-disease models persists [5,17]. The issue is much more pronounced for parameters used to formulate human response (e.g., human response sensitivity to reported death) than to formulate the spread of the disease (e.g., incubation period). There are several reasons for this challenge. First, while laboratory experiments can provide inputs about disease-related model parameters, we often lack such a luxurious input for behavioral parameters. Second, human response is lagged. For disease-related parameters, effects are generally observed within a week or so (e.g., the delay between exposure, symptom onset, and recovery or death), while behavioral parameters (e.g., changes in public risk perception) take longer, slowly going through several information filters such as public media, social media, and daily conversations. Third, behavioral parameters often vary across different regions and demographics, whereas most disease-related parameters (such as the distribution of incubation period) are similar across different populations. The implication is that the estimated behavioral parameters from one region may not be easily transferable to another setting. These issues introduce several challenges for behavioral epidemic modeling, inhibiting model validation, reliable policy analysis, and accurate projections.

In this study, we systematically examine the challenges of parameter estimation in a specific type of behavior-disease model. Our primary focus is on addressing parameter identifiability challenges, commonly referred to as an inverse problem or model identifiability, rather than developing new models or theories regarding human behavior in epidemic models. Furthermore, behavioral mechanisms can be modeled in various ways and can represent different phenomena. Our focus here is on one specific mechanism, referred to as risk-response [9]. When modeled endogenously, the risk-response feedback loop represents societal response to change in risk perceptions and influences contact rate or infectivity. This is one of the most common additions in behavior-disease models.

From the existing literature, we select a previously validated behavioral epidemic model that represents risk-response endogenously while having a small number of parameters to be estimated jointly. This model, known as the SEIRb model (*b* stands for behavior), is a simple extension on SEIR models and formulates infectivity rate as an inverse function of recent death to represent human response [2]. The addition of behavioral feedback in the SEIRb model has been shown to substantially enhance forecasting performance of COVID-19 models [2,9].

Using the SEIRb model, we generate synthetic data with a set of assumed parameter values as ground truth. We design experimental setups in which the ground truth (true values of parameters used to generate synthetic data) is concealed from the researcher. Our objective is to evaluate the accuracy of parameter recovery and identify any unique challenges in estimating behavior parameters compared to disease parameters.

## 2. Background

### Behavioral epidemic models

Epidemic models date back to the works of Kermack and McKendrick [13] and have been successfully applied to a wide range of infectious diseases [18]. In many subsequent epidemic models since this foundational work, human behavior, or infectivity rates, remain constant or change by external factors [2]. Behavioral epidemic models represent a departure from traditional infectious disease models in that they capture the interdependence between the state of the disease and human behavior [2, 16]. The primary premise of the behavioral epidemic models revolves around capturing change in human behavior within epidemic models.

There are several systematic literature reviews studying different approaches to incorporate human behavior in epidemic models [11,12,19]. One common classification of past efforts is based on modelers’ approach to formulate change in behavior and specifically the source of information that drives behavior change in the models. In reality, such information can be widely available through media or public awareness campaigns [20–22], or it may come from local networks like neighborhoods and friends [23–25]. Another model classification considers how behavior change is formulated. Some models implement exogenous behavior change, controlled by external factors or data input [26–29]. In this approach, change in behavior is not a function of the models’ state variable, but is assumed based on the modelers’ intuition or occasionally feeding time series data. On the other hand, many modelers incorporate behavior change endogenously, where human behavior changes within the model and in response to the system’s states [2,15,30]. This latter endogenous approach is essential in capturing potential future changes in human behavior and leads to developing models in which behavior and disease dynamics are coupled.

Among the endogenous behavioral epidemic models, a critical classification lies in identifying the driving factor behind behavior change. In most of the models, behavior changes in response to disease prevalence (infections or mortality) [31–33]. The core idea is that as the disease spreads, people perceive risks, and react by increasing their NPI adherence, which decreases the spread of the disease. There are also studies where change in human behavior is more explicitly modeled by representing opinion dynamics or the spread of fear coupled with the spread of the disease [34–36].

We narrow our focus to a specific type of behavioral epidemic models. We look into a model that incorporates endogenous societal risk-response, where responses change with perceived risks represented by the recent mortality rate. Referred to as SEIRb, this is one of the simplest yet effective approaches to incorporate change in human behavior in epidemic models [2]. In these models, transmission rates dynamically change in response to society’s perceived risk of deaths. An increase in deaths elevates risk perception, leading to a reduction in transmission rates through the adoption of NPIs. As more people adopt NPIs, disease transmission and the subsequent death rate decreases lowering risk perception. This creates a balancing feedback loop between disease mortality and behavior change. This feedback loop has been shown to be pivotal in the long-term predictive power of multi-wave COVID-19 trajectories [2].

### Parameter estimation

In disease transmission models, it is a common practice to estimate epidemiological parameters that cannot be directly observed by fitting a model to disease data such as infected cases, deaths, hospitalizations, and mobility. Whether or not model parameters can be reliably estimated by this practice is a topic of model identifiability research. For example, when the model is identifiable, infectivity rate (often shown by β) can be estimated by finding a value that minimizes the difference between data and simulation, measured by sum of squared errors. Prior to the COVID-19 pandemic, only a few behavior-disease models utilized real-life data for parameterization or validation [11,12,19]. While the pandemic has spurred the development of data-driven models, to the best of our knowledge, none have studied the topic of identifiability.

Parameter identifiability tests are typically recommended as the first step in parameter estimation to ensure reliable estimation of model parameters from observed data [37–39]. These tests take two forms: structural identifiability examines parameter estimation under ideal conditions, and practical identifiability examines realistic conditions of noisy and limited data and improper model structures [39–41]. Parameter identifiability of SIR models with latency, seasonal forcing, immunity, and various other features are extensively covered in epidemic literature [42–44]. However, there is a notable gap for parameter identifiability in behavioral epidemic models. This shortage of theory hinders accurate parameter estimation in these models.

The challenge of parameter identifiability in coupled behavior-disease models arises from increasing model complexity from the inclusion of more compartments, variables, and feedback relationships to describe the behavior side of the model [16]. The uncertainties and biases accompanying parameter identifiability escalate as both the number of jointly estimated parameters and the model’s complexity increase [42,45].

Even for simpler models, due to computational complexities and mathematical challenges, modelers tend to bypass parameter identifiability analysis [39,43]. Instead, they proceed directly to fitting models to available data, employing methods such as least squares fitting [46], maximum likelihood estimation [47], or Bayesian estimation [48], then measure how well simulation results fit data [38,49]. This approach might not significantly impact modeling outcomes because researchers are already cognizant of identifiability challenges when estimating disease parameters in SIR-like models. However, the same cannot be asserted when modelers must estimate both disease and behavior parameters.

Hence, our study designs practical experiments to systematically investigate the challenges of parameter estimation in behavioral epidemic models. As stated, we focus on models with endogenous societal risk-response where disease and behavior parameters must be jointly estimated. We use the SEIRb model to generate synthetic data and then try to uncover model parameters using a simplistic, yet commonly employed, least squares approach. We compare the accuracy of parameter estimation, the model’s fit to data, and its prediction accuracy across different stages of the pandemic.

## 3. Study hypotheses

We offer three major hypotheses related to delay in behavioral response, neglecting behavioral response, and availability of behavior data.

### Effect of a delayed risk-response

Studies have shown that disease parameters in SEIR models are structurally identifiable [44,50]. This means that under ideal conditions where modelers have accurate disease data (e.g. cases and deaths) and the model structure closely represents reality, one can accurately estimate parameters. In models with endogenous societal risk-response, both behavioral and disease parameters need to be jointly estimated. One can assume that if the disease parameters are identifiable with accurate disease data and model structure that closely represents reality, the same extends to behavioral parameters. Thus, we present the following hypothesis:

*H1a: In the presence of an accurate model and disease data*, *disease and behavior parameters can be jointly estimated throughout the pandemic accurately*.

While we intuitively expect the above to hold, one may argue that early in the pandemic people have not reacted to the state of the disease and are still learning about the situation. Thus, the available data, even if accurate, does not fully incorporate human sensitivity to possible change in risk. If that holds, H1a will not be fully supported. The implications are that the disease-related parameters would be estimated accurately earlier in the pandemic but for behavioral parameters a longer time series will be needed. As more data are collected over the course of an outbreak, estimates for behavioral parameters should also become more precise, and uncertainty should decrease. Thus, we propose to investigate the following competing hypothesis:

*H1b*: *Estimation of behavioral parameters is unreliable before the peak of the first wave of the pandemic even with accurate model and disease data*.

### Effect of neglecting risk-response in an epidemic model

All models are simplified representations of the real world, but still having relevant and reasonable assumptions are crucial for accurate parameter estimation [51, 52]. Structural uncertainties arise when models include unrealistic scenarios or fail to consider essential aspects of the outbreak, leading to inability of the model to replicate the disease trajectory [53, 54]. Such uncertainties cannot be eliminated by refining data; instead, model assumptions that are responsible for failure must be corrected [42]. To test the importance of the risk-response assumption, we use a conventional SEIR model that lacks behavioral feedback to estimate parameters from a dataset produced by a behavioral model with endogenous risk-response. This is technically a structural sensitivity test [55] and represents the situation where a modeler neglects behavioral phenomena of a system by using a model that lacks such mechanisms. If supported, the implication is that even if extensive data are available, a model that is not properly representing behavioral responses will fail in parameter estimation and, consequently, in projection. Thus, we propose this hypothesis:

*H2*: *Neglecting risk-response leads to unreliable parameter estimation*, *even with extensive data*.

### Effect of integrating disease and public behavior data

Various data sources can be used to validate a coupled model. Specifically, a coupled model that generates disease data endogenously can be compared with the pandemic data for validation and parameter estimation, and it is expected that such models should also generate risk response accurately even if they are not compared with risk perception data. Numerous studies have explored data types that can serve as proxies for behavioral responses during outbreaks within target populations [56,57]. Despite this, coupled behavior-disease models that have estimated parameters from data often have focused on minimizing the difference between the disease data and simulation, without trying to replicate the behavioral data as well [15]. Moreover, data on risk perception, or change in mobility, or any other behavioral reaction is argued to be critical for further model validation (refer to the topic of partial model tests by Homer [58] and Oliva’s discussion on the importance of partial model tests [59]). Availability of such behavioral data is hypothesized to significantly improve the estimation of behavior parameters and increase the accuracy of disease forecasting [10–12,19,24].

Building on these ideas, one can assume that if all data, including data on human behavior such as mobility, are observable and accurate, and model structure closely represents reality, then joint utilization of disease and behavior data for parameter estimation should improve parameter estimation accuracy. Thus, we investigate the following hypothesis:

*H3: In the presence of an accurate model and data*, *the addition of public behavior data improves accuracy of parameter estimation throughout the pandemic*.

Based on the above hypotheses, we also propose that, with better parameter estimation, the accuracy of model projections will improve throughout the pandemic if a model structure with assumptions that closely align with reality is used and the estimation process incorporates public behavior data in addition to accurate data on the state of the disease (e.g., daily death).

## 4. Method

### 4.1. Procedure

We use a three-step process to explore the challenges of parameter estimation under various experimental conditions. First, we create synthetic data from a model with assumed parameter values to serve as “ground truth.” Second, in an inverse process, by providing a fraction of the data, we try to estimate the assumed parameter values through model calibration. By comparing the estimated parameter values from the second step with the ground truth values that were used in the first step to create the synthetic data, we evaluate the accuracy of the estimated parameters. This process is repeated for different conditions to examine uncertainties in parameter estimation for different delays in behavioral response at different periods of the pandemic (H1), when considering incorrect model assumptions (H2) and when utilizing data on public behavior (H3). Finally, to evaluate the potential significance of errors made during calibration, we compare death projection with the estimated parameters with the ground truth data for a period of 365 days.

### 4.2. Synthetic data generation

We use a stochastic SEIRb model with assumed parameter values to generate synthetic data. SEIRb builds upon the traditional SEIR model by incorporating a balancing feedback loop to capture behavioral responses endogenously [2]. In short, the model is consistent with the SEIR model and adds dynamic human response to risk; the fact that an increase in the death rate can lead to an increase in risk perception, resulting in more non-pharmaceutical intervention (NPI) initiatives and a decrease in cases.

We make slight modifications to the SEIRb model to make our experiments more realistic. We include autocorrelated noise affecting exposure rate to depict intrinsic stochastic factors that cause real-world data to deviate from expected values such as weather patterns and holiday movements [60]. While the stochasticity can be introduced to other parts of the model too, such as directly to death, mathematically, we do not expect any meaningful difference with the current formulation as all stochastic elements can be summed up in one variable. This noise is derived from exponential smoothing of white noise, with a mean of 1, standard deviation of 0.3, and a correlation time of 15 days. The correlation time reflects how past noise values influence the current value. It follows an exponential decay, with the time constant matching the correlation time (refer to the topic of correlated noise in [61]).

**Fig 1:**
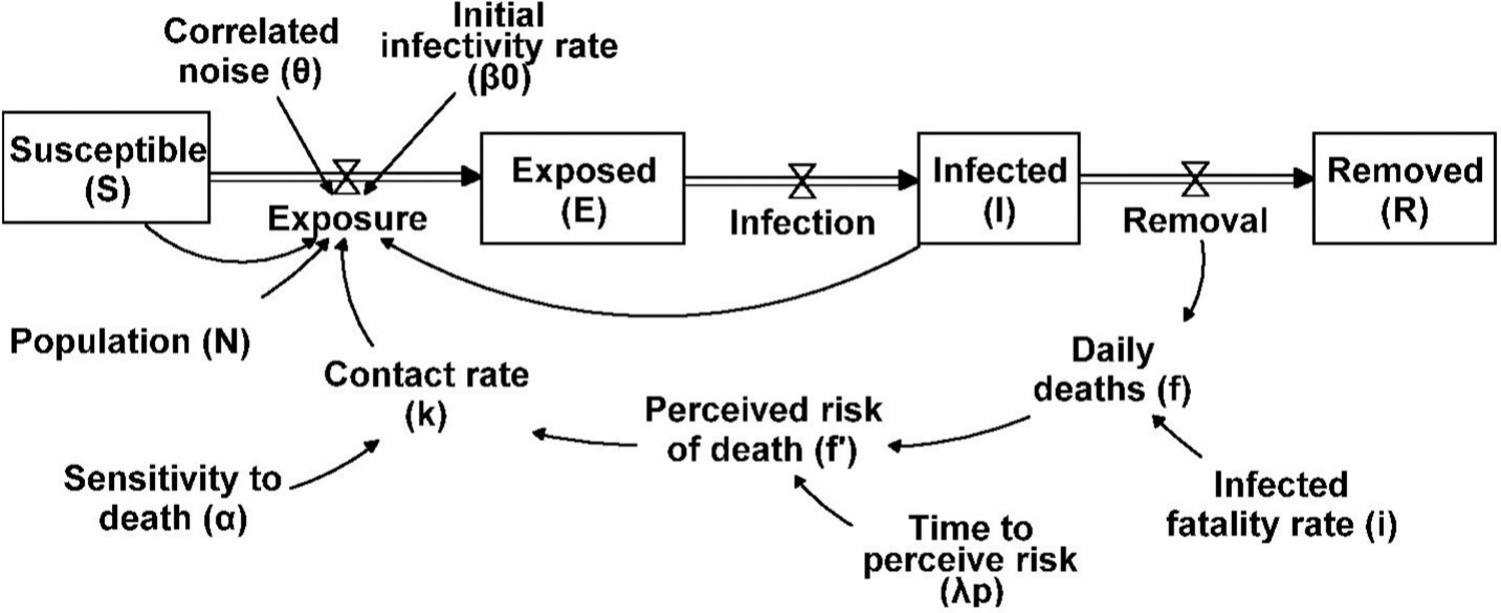
A simple representation of the SEIRb Model with an additional stochastic variable.

Fig 1 depicts the model structure. See S1 Text for detailed model equations.

Our method does not aim to replicate any specific disease progression with synthetic data. However, we employ parameter values within realistic ranges associated with COVID-19. S1 Text contains a list of the parameter values used in generating the data. The model generates two major outcomes of daily deaths and contact rate which we use to estimate model parameters. The contact rate serves as data on public behavior, and it represents observations of how people are adjusting their contacts during the progression of the disease. We assume that both daily deaths and contact rate are precisely measured.

**Fig 2:**
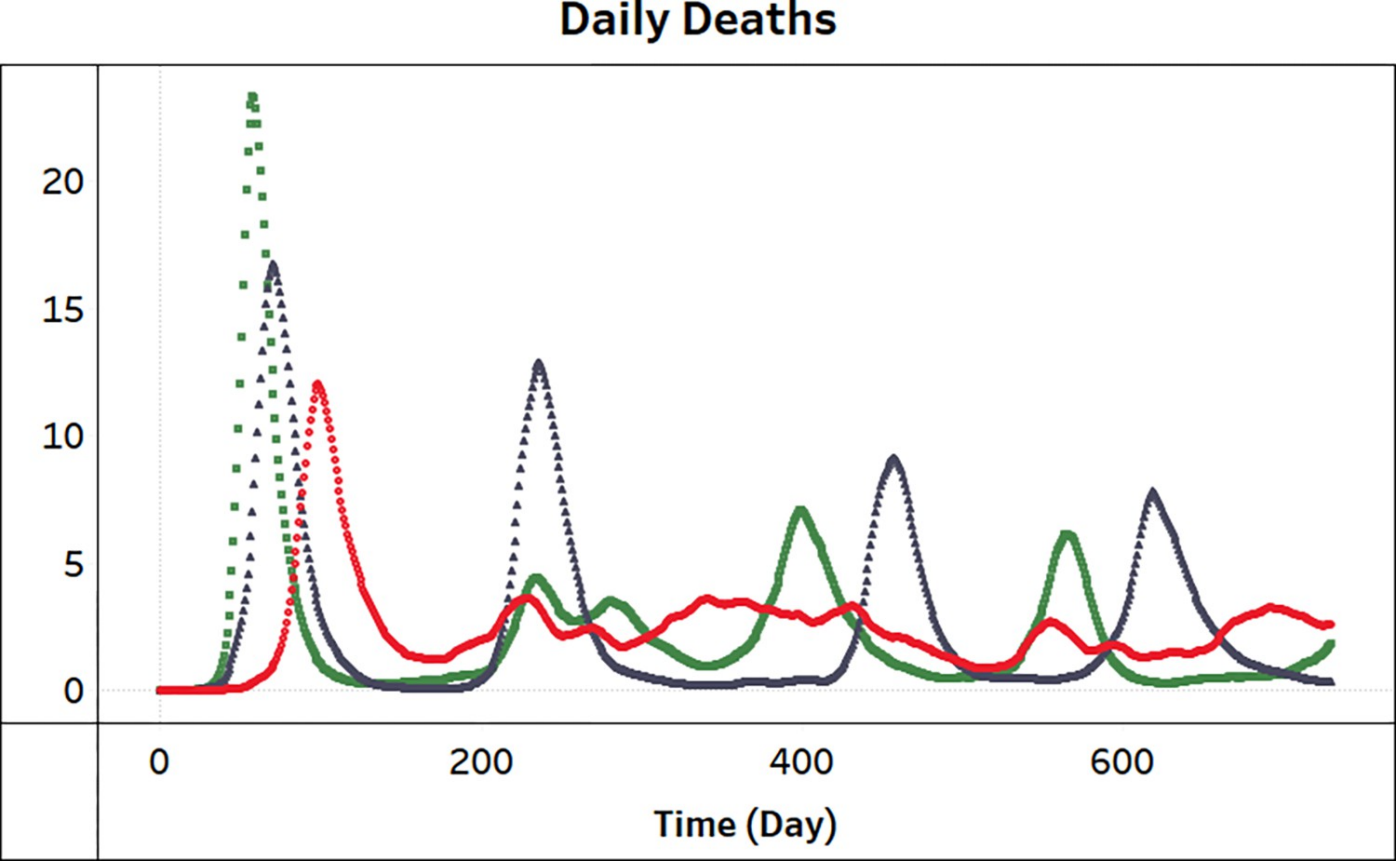
A sample of three simulation runs (synthetic daily death data) from the same model with the same parameter values but with different realizations of stochasticity over 730 days.

To generate synthetic data of daily death and contact rate, we simulate the SEIRb model using 100 noise seeds. This results in 100 stochastic realizations of both daily death and contact rate. Fig 2 shows a sample of simulation outcomes for daily death.

### 4.3. Experimental conditions

For all experimental setups, only a portion of data is utilized to estimate parameter values. Specifically, the available data is either from the first 60 days of the pandemic (early stage), 120 days (following the first wave), or 365 days (after one year). This design helps us investigate our hypothesis at different periods of the pandemic.

First, we use a “perfect model” (SEIRb, the deterministic version of the same model that created the synthetic data) and daily deaths data to perform model calibration. We record the estimated parameters for each of the different data periods.

Second, we repeat the first experiment using an "imperfect model" (SEIR, a model that does not account for human behavior response). This allows us to determine the impact of using a better model for parameter estimation.

And finally, we perform model calibration using the "perfect model" (SEIRb) but also incorporating both daily deaths data and public behavior data represented by contact rate. This enables us to investigate the benefit of utilizing data on public behavior in improving model calibration for various periods of the pandemic.

The entire process for each experiment is summarized in Table 1.

**Table 1:** Testing three major hypotheses through 9 calibration experiments.

Hypothesis	H1: Effect of delay in behavioral response	H2: Effect of neglecting behavioral response	H3: Effect of data on public behavior
Data for calibration	Daily deaths	Daily deaths	Daily deaths + contact rate
Model for calibration	SEIRb	SEIR	SEIRb
Calibration period	Early (60 days), Mid-term (120 days), Late (365 days)	Early (60 days), Mid-term (120 days), Late (365 days)	Early (60 days), Mid-term (120 days), Late (365 days)

### 4.4. Parameter estimation process

We use the synthetic data generated, depending on the experimental condition being evaluated, to estimate the model parameters. We consider three parameters of the model to be unknown and grouped into two sets: the "disease set" consisting of the infectivity rate (β_o_) and the "behavior set" consisting of sensitivity to risk (α) and time to perceive risk (λ_p_). In Experiment 2, where we examine the effect of neglecting behavioral response using the "imperfect model" (SEIR), we only need to estimate the infectivity rate (β_o_).

Parameter estimation is done using a calibration process that involves minimizing the difference between model output and data [59]. Detailed information about the parameter estimation method is provided in S1 Text. Since we have 100 separate outcomes of data, each experiment results in 100 estimates of each unknown parameter. For each experimental setup, we compare results obtained from different periods (early 60 days, mid-term 120 days, and late 365 days) and then compare all three experimental conditions.

We assess the performance of parameter estimation by comparing the estimated values to the true parameter values, in terms of bias (closeness to the true value) and variance (dispersion of estimates around the median). A good estimation performance is characterized by low bias (with the median of the estimates close to true values), and low variance (with most estimates tightly clustered).

### 4.5. Deaths prediction

Using the estimated parameter values, we generate simulated deaths data for 365 days into the future using the estimated parameters obtained from each experiment. The purpose is to see to what extent the estimation errors in parameters affect projection performance. The prediction error is calculated as the daily mean absolute percentage error between simulated deaths and actual deaths cumulated over 365 days (see S1 Text for equation).

### 4.6. Sensitivity analysis

We test the robustness of our findings through several sensitivity analyses. Specifically, first we examine the sensitivity of our results to change in the delay period in human responses to risk. To that end we test a wide range of perception delay values from 20 to 200 days. Then, we conduct a set of tests to examine identifiability in the presence of three additional mechanisms: seasonality, variant emergence, and loss of immunity after infection. This specific test helps to see if oscillations generated from behavior can be distinguished from other oscillatory mechanisms. Third, we increase the number of unknown disease parameters adding to the complexity of the process of parameter estimation. Fourth, we conduct experiments with different parameter values expanding the range of tests. Finally, we examine the sensitivity of the results to the extent of available behavior data by testing the incremental impact of adding more behavior data. The results are summarized in the results section and reported in detail in S1 Text.

## 5. Results

### 5.1. Effect of delay in behavioral response on parameter estimation

**Fig 3:**
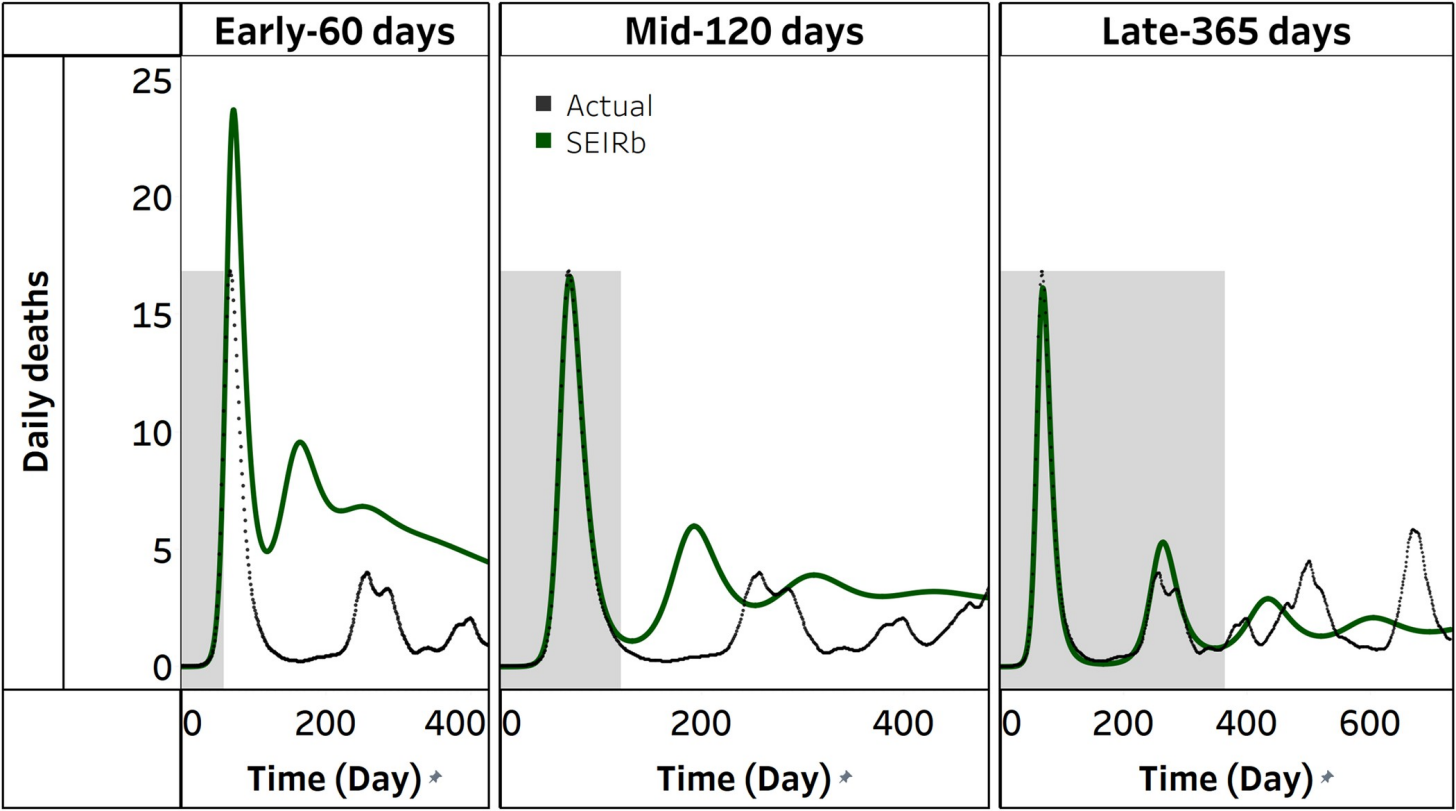
Actual vs simulated deaths from SEIRb model calibration for a single stochastic dataset. The shaded area is the area for which data were available, and the border between shaded and unshaded area is the time for model calibration (observation time). The unshaded portion is projection for the next 365 days.

Before analyzing the results from calibration of 100 different realizations of data, we focus on one single estimation as an example. Fig 3 compares actual daily deaths with simulated deaths from one calibration outcome of the SEIRb model. Simulated deaths accurately replicate data for all stages of the disease outbreak during the periods for which data are available. However, during the projection period, calibration in the early stage leads to poorly fitted outcomes. The fit improves in the mid stage and is best at the late stage.

**Fig 4:**
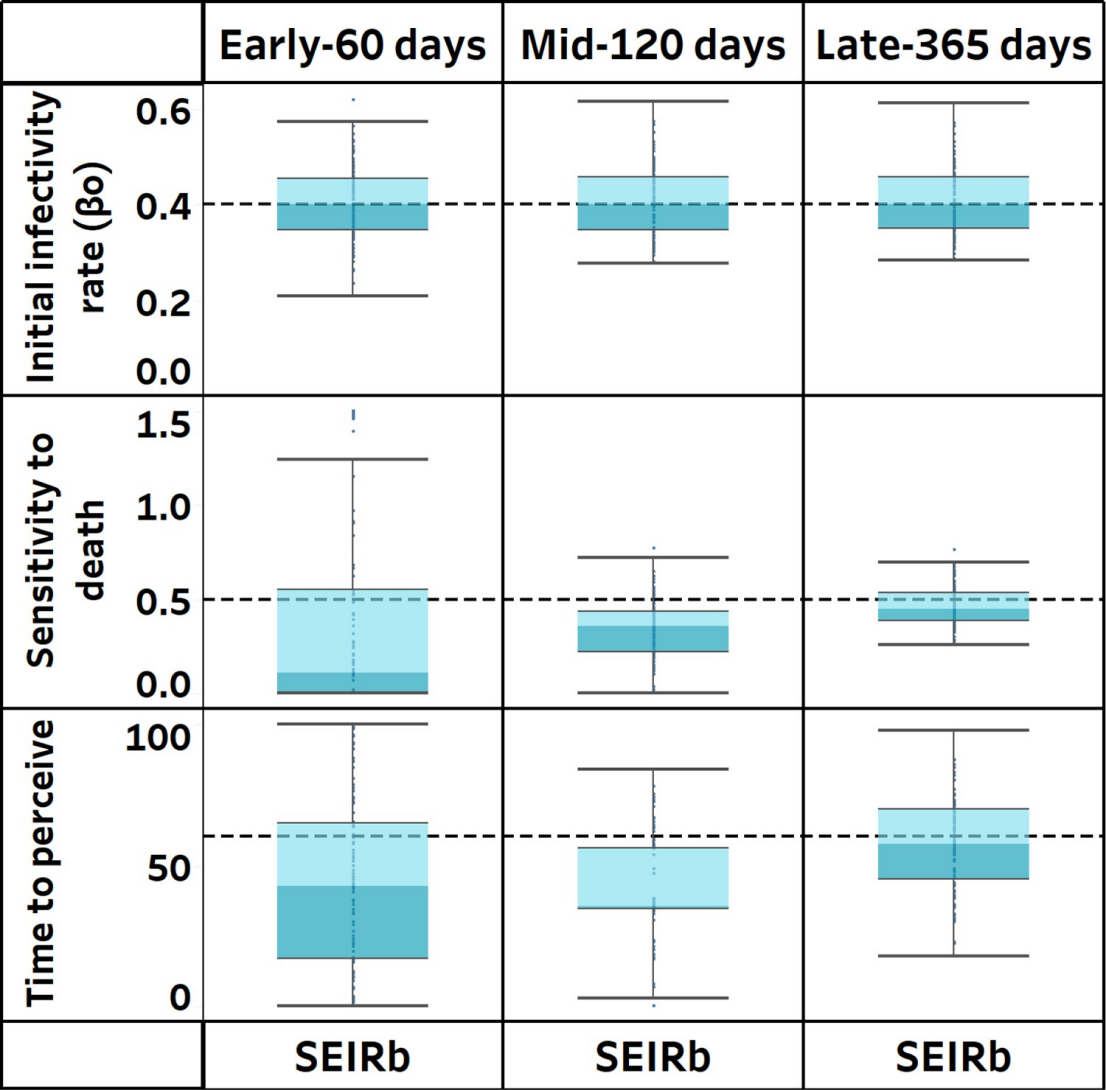
Estimated parameter values using only deaths data to calibrate the SEIRb model. Actual parameter values are indicated by broken lines.

Next, we systematically compare results from calibration with the ground truth values i.e., the assumed values of the parameters during data generation. Fig 4 illustrates the distribution of parameter estimates from calibrating the SEIRb model with daily deaths data. Fig 4 shows that efficient estimation of β_o_ is achieved with low bias and variance at all stages of the outbreak. In contrast, estimation of behavior parameters (sensitivity to death and time to perceive) is unreliable with high bias and variance in the early days of the outbreak but improves at later stages.

We then statistically compare the estimation errors during different stages of the outbreak (see S1 Text). The estimates of β_o_ (Infectivity rate) remain relatively consistent, with no significant difference in errors between earlier and later stages of the outbreak. On the other hand, estimation errors of behavior parameters (sensitivity to death and time to perceive) are significantly higher in earlier stages of the outbreak compared to later stages (p<0.001), indicating less accuracy in the former. Overall, the smallest parameter estimation error was achieved using 365 days of data, which explains the better fit of simulated deaths to actual deaths obtained with this observation time.

We conclude that even when using the same model that generated the data, efficient parameter estimation could not be achieved in the early days of disease outbreak for behavioral parameters. Interestingly this is not the case for the disease-related parameter, infectivity rate. Thus, researchers with enough accurate disease data can estimate the initial reproduction rate of R_0_. By not finding support for H1a, we note that the challenge of estimating behavioral parameters during the early periods of the pandemic is beyond data and model accuracy.

Overall, the findings suggest that even a "perfect" model requires data that includes at least one wave of the disease to achieve accurate behavior parameter estimation, and by extension, a good model fit. As the estimations improve after the first wave, we can argue that the difficulty of parameter estimation arises from the delay in public perception and reaction to the changes in the state of the disease.

### 5.2. Effect of neglecting behavioral response on parameter estimation

**Fig 5:**
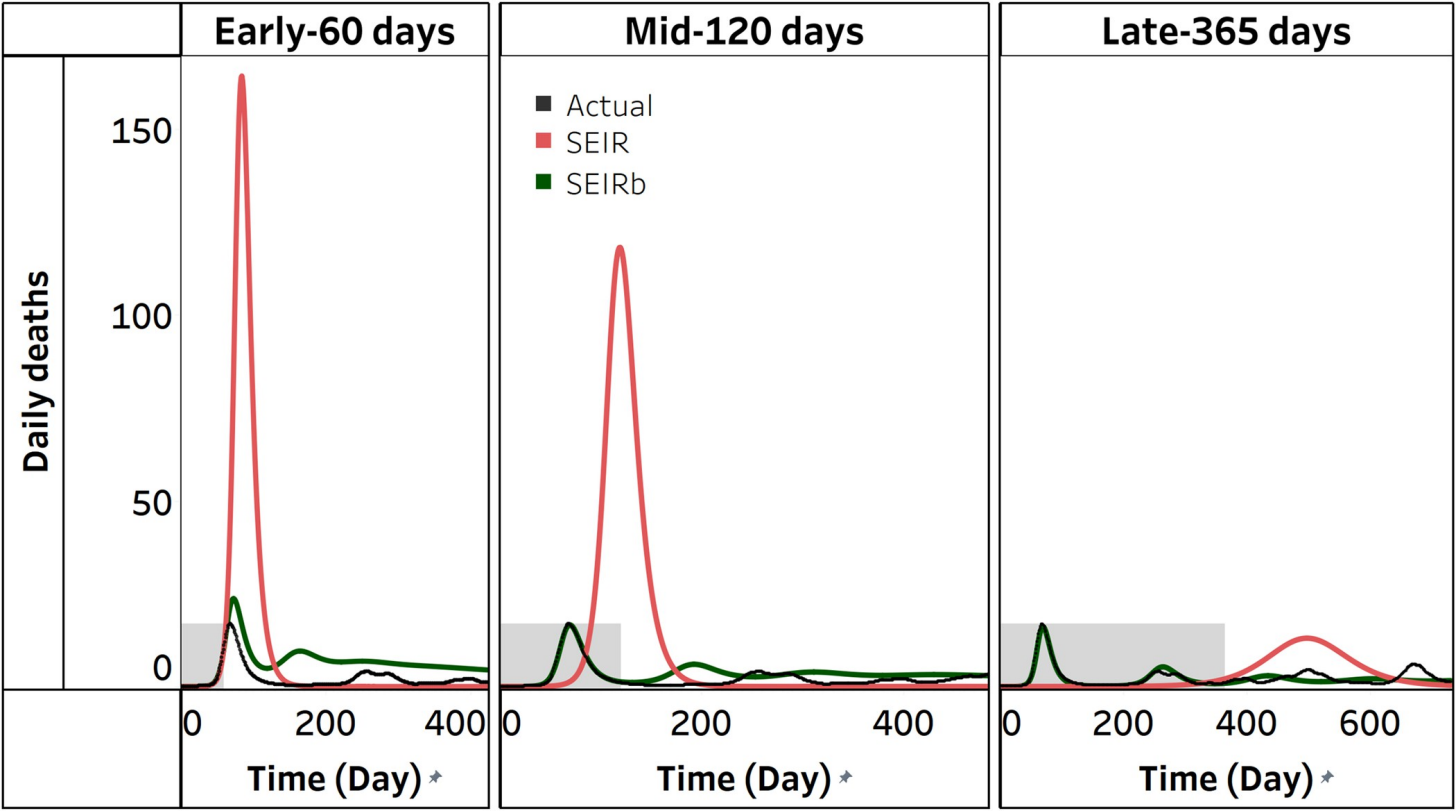
Actual vs simulated deaths from the SEIRb and SEIR. The shaded area is the area for which data were available, and the border between shaded and unshaded area is the time for model calibration (observation time). The unshaded portion is projection for the next 365 days.

Would using a model that insufficiently represents human behavior provide reasonable projections? We start by analyzing a single simulation run. Fig 5 compares actual deaths with simulated deaths from calibrating the SEIRb and SEIR models with daily deaths data. Simulated deaths from the SEIR model only fit well with actual data in the early stage of disease outbreak, and only for the period that data are available (note early 60-days, depicted by grey area in Fig 5). However, during the projection period, the SEIR model fails to replicate the oscillations in actual deaths data and produces a single, overestimated wave. As more data are used for calibration, the overestimation decreases, but the wave lags significantly, and simulation does not match data. This behavior is consistent across all SEIR calibration outcomes.

**Fig 6:**
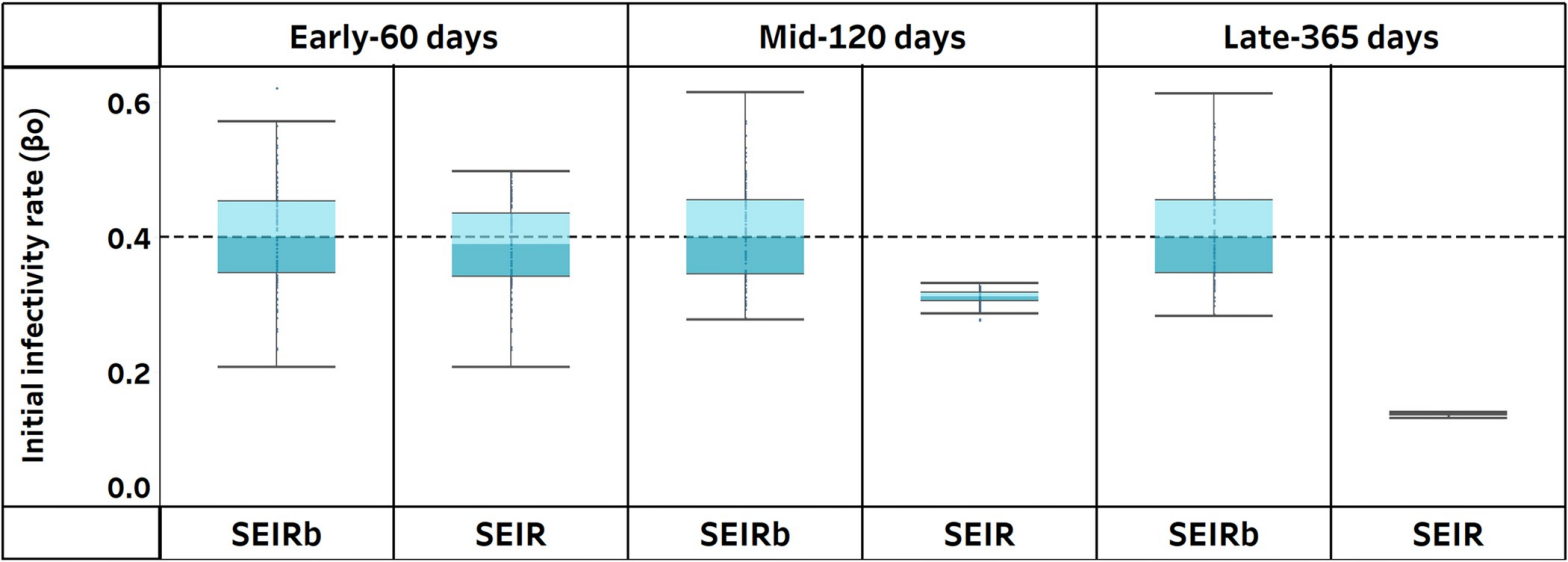
Estimated parameter values using only deaths data to calibrate the SEIRb and SEIR models. Actual parameter values are indicated by broken lines.

Next, we systematically investigate, over all 100 realizations, the effect of using an improper model structure on the accuracy of estimated parameters. Fig 6 displays the distribution of parameter estimates obtained from calibrating the SEIR model.

Interestingly, the improper SEIR model yields comparable β_o_ estimates to those of the SEIRb model in the pandemic’s early stage. The SEIR model produced more accurate estimates, with a smaller β_o_ estimation error (p<0.001; see S1 Text). However, as calibration periods lengthen, β_o_ estimation accuracy declines with the SEIR model. The estimates become more precise but significantly deviate from true values. This suggests underfitting and explains why the SEIR model fails to capture the data in the long run.

Overall, we find support for H2 that even with extensive data, and even for non-behavioral parameters (such as infectivity rate), parameter estimation would be unreliable if models that neglect behavioral response, such as SEIR, are used. This stresses the fact that data availability when models are not proper for the purpose of analysis will not help. The fact that early in the pandemic, the disease parameters might be accurately estimated with an improper model (SEIR) raises concerns that such early estimations can mislead modelers to rely overconfidently on such models for a long period before time eventually proves that such models substantially fail to represent the reality.

### 5.3. Effect of data about public behavior on parameter estimation

**Fig 7:**
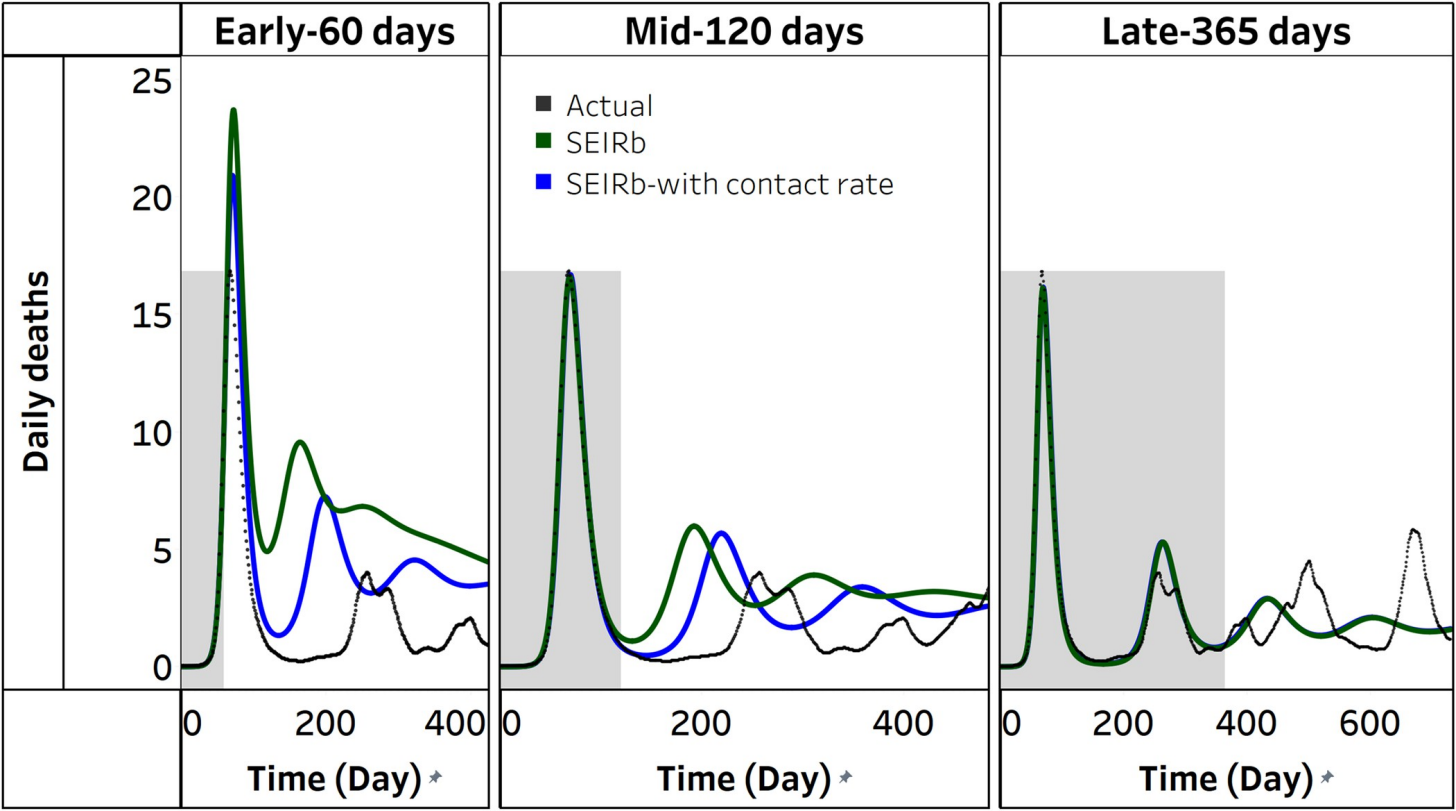
Actual vs simulated deaths from the SEIRb model with and without data on public behavior. The shaded area is the area for which data were available, and the border between shaded and unshaded area is the time for model calibration (observation time). The unshaded portion is projection for the next 365 days.

Can availability of additional data on public behavior help? We first check a single calibration effort as an example. Fig 7 compares actual daily deaths with simulated daily deaths from calibrating the SEIRb model with and without the use of contact rate data. Irrespective of the use of contact rate data, simulated deaths effectively replicate actual deaths in the period for which data is available. Differences in fit to data emerge when projecting deaths for the next 365 days. Fig 7 shows that using contact rate data results in better fit to data in the early stages of disease outbreak, with differences in fit becoming less apparent in the mid-stage, and negligible in the late stage. These findings apply to all 100 calibration outcomes.

**Fig 8:**
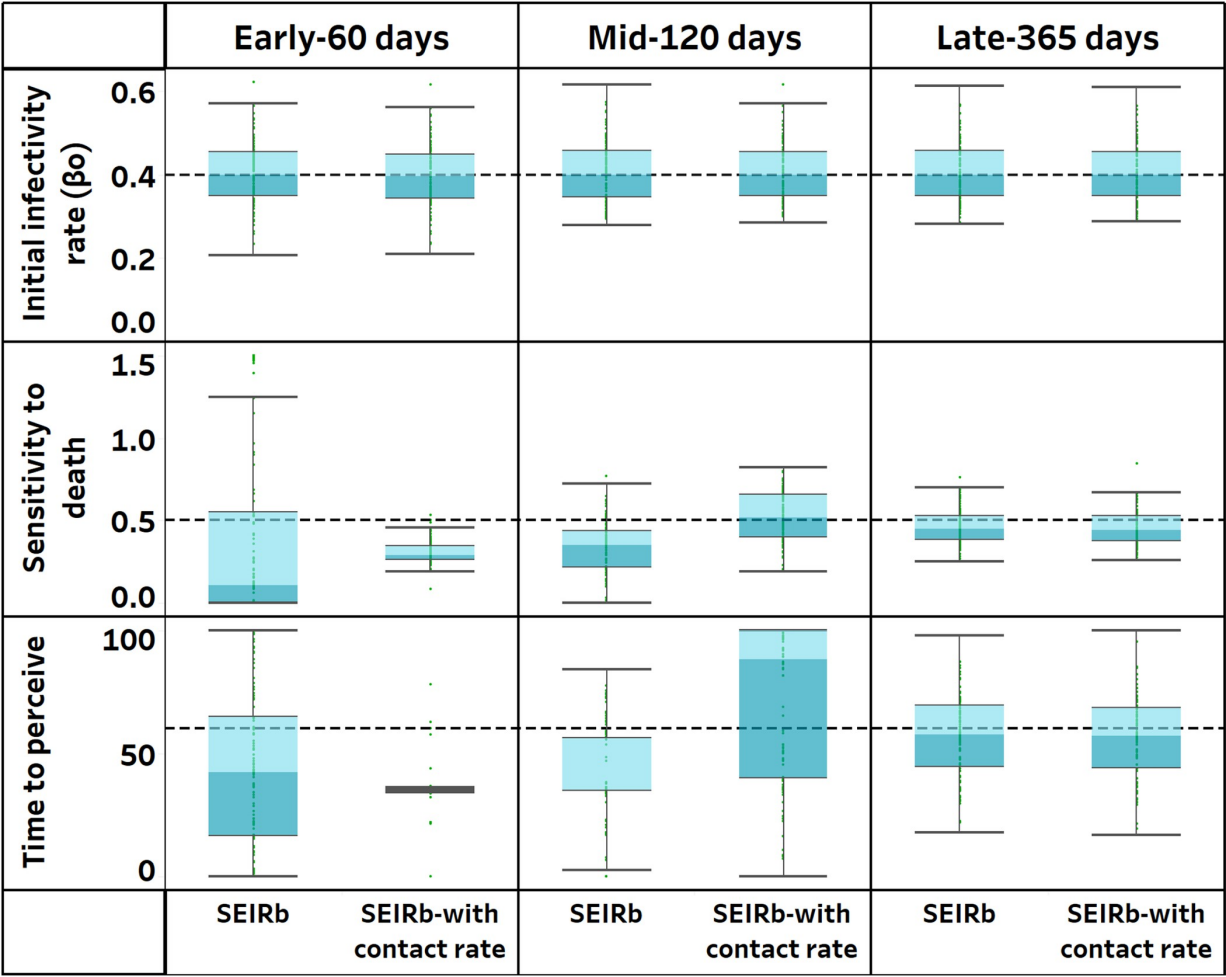
Estimated parameter values with and without using behavior data to calibrate the SEIRb model. Actual parameter values are indicated by broken lines.

Fig 8 displays the distribution of parameter estimates obtained from calibrating the SEIRb model with and without the use of contact rate data.

We observe that the accuracy of behavior parameter estimation improves over time with the incorporation of contact rate data in model calibration. Early stage estimates of behavior parameters appear more precise when contact rate data is used than otherwise. However, only mid-stage estimates of sensitivity to death had improved estimation accuracy. In late stage calibration, no notable difference is observed when contact rate data is used.

Additionally, the estimation errors for behavior parameters are significantly smaller in the early stage (p<0.05) when contact rate data is used, and estimation errors for sensitivity to death are significantly smaller in mid stage calibration (p<0.001) with the use of contact rate data. In late stage calibration, the inclusion of contact rate data does not yield significant differences in estimation errors (see S1 Text).

Overall, these findings partially support H3, suggesting that using data on public behavior improves accuracy of parameter estimation. This effect is most pronounced during the early stages of pandemics, when disease data is insufficient in capturing the impact of behavioral responses to the disease outbreak. While we see statistically an improvement in estimation, Fig 8 shows that the values are still far from the ground truth.

### 5.4. Variability in model trajectory

**Fig 9:**
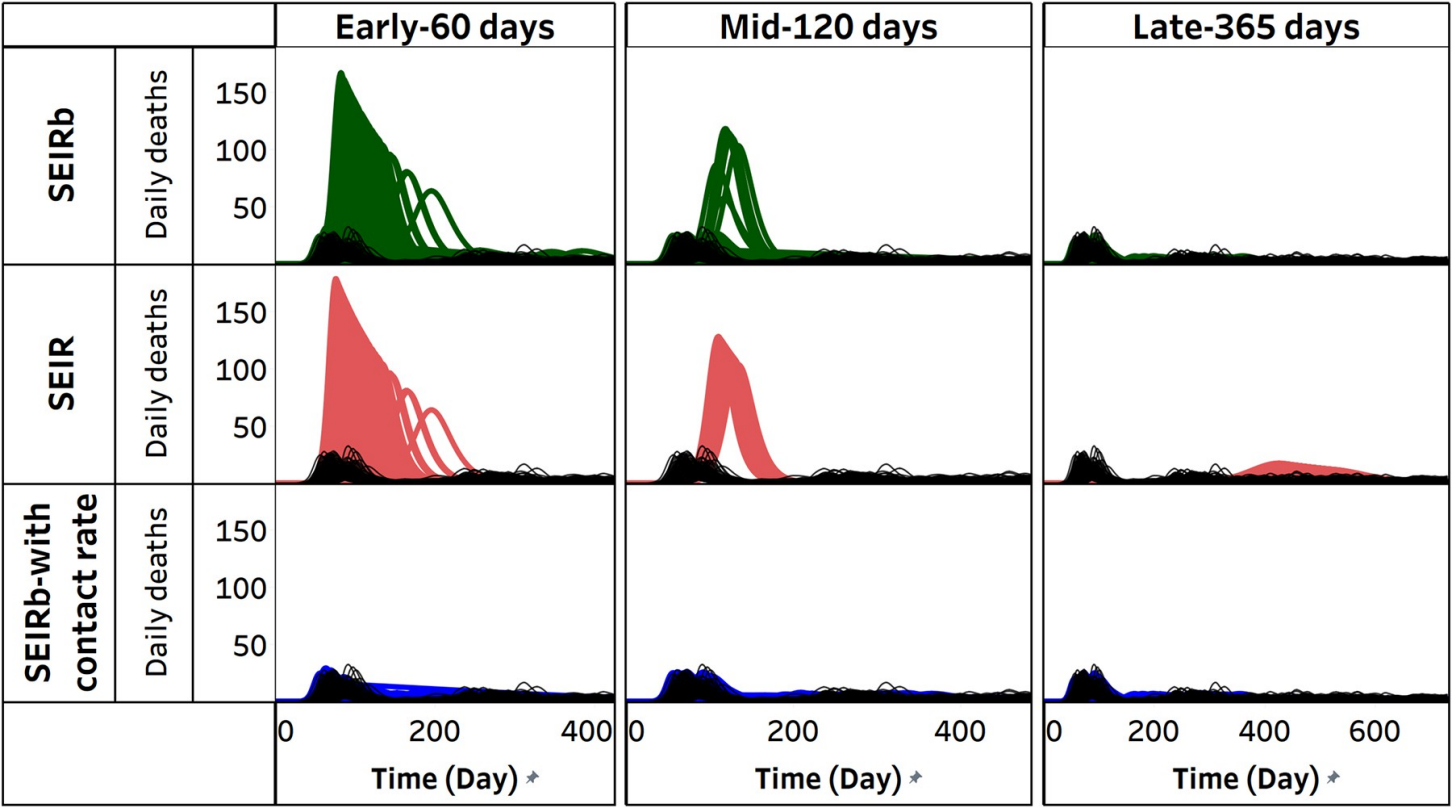
Actual vs simulated deaths from all experiments. The black lines are 100 synthetic deaths data used for calibration. Green, red, and blue lines are 100 simulated deaths from calibrating the SEIRb model, SEIR model, and SEIRb model with contact rate data, respectively.

In order to compare projections across different experimental conditions, Fig 9 depicts all individual simulations. We see that model fit improves over time for all conditions examined. However, when behavioral response is disregarded in the SEIR model, the model fit is notably the poorest, as it fails to replicate the oscillatory trends observed in the data. For early-stage projections, utilizing contact rate data with a perfect model yields the most favorable results. On the other hand, for late-stage projections, having a perfect model alone is sufficient.

### 5.5. Future death rate projection

**Fig 10:**
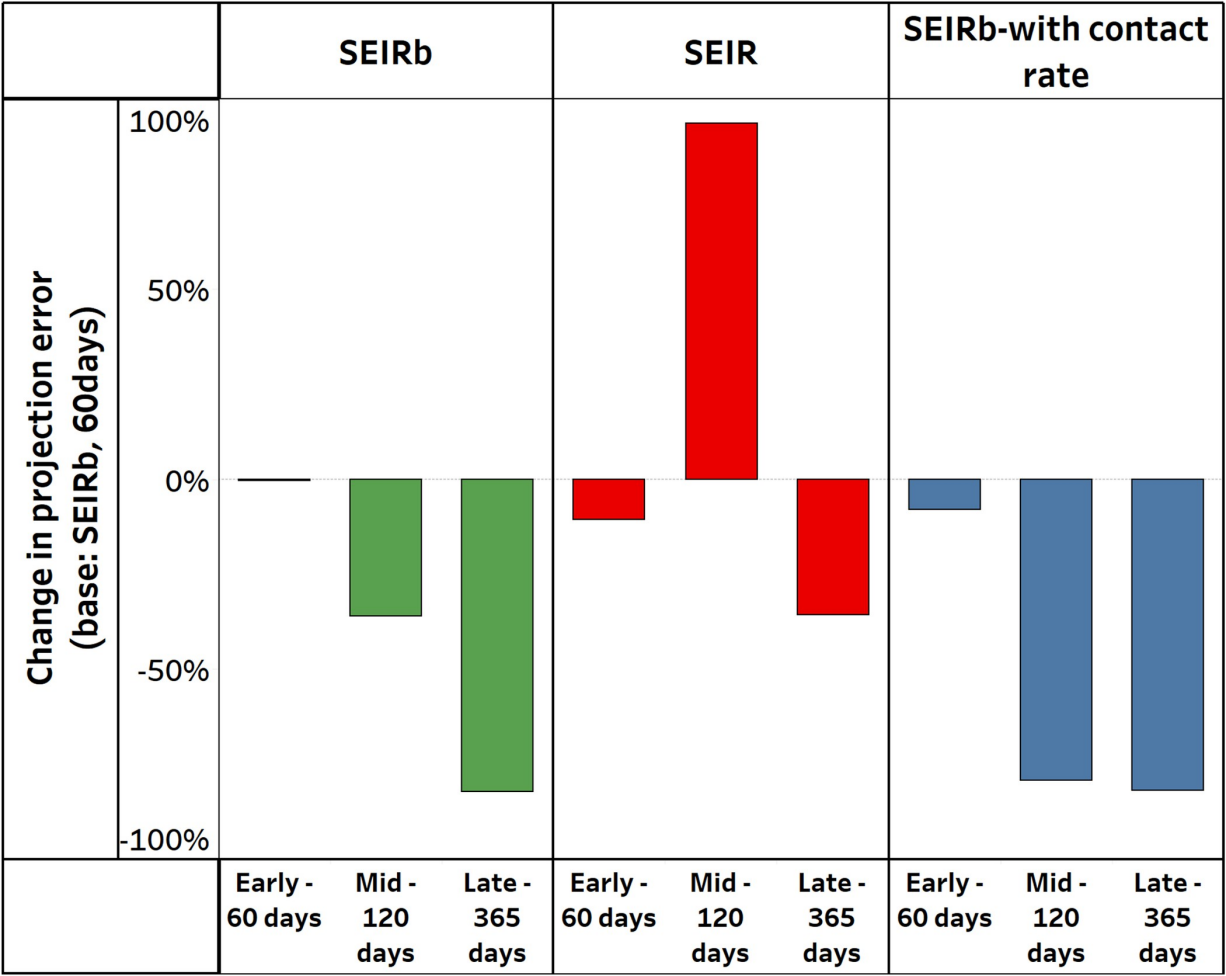
Change in cumulative errors for next 365-day death projection. All values are normalized to the cumulative error of early-60 days calibration of the SEIRb model i.e. the first bar on the left is 0%.

If the purpose is outbreak projection, a reasonable question is how much does the estimated errors in parameter values matter? Fig 10 shows the change in cumulative errors in the next 365-day death projection of different models and data availability conditions from the previous stage. All values are normalized to the projection error of an SEIRb model that uses the early 60-day data (i.e., the first bar on the left is at zero percent).

Fig 10 shows that calibrations utilizing the SEIRb model demonstrate a decreasing trend in projection errors over time. These results indicate that, given accurate data and model assumptions, prediction accuracy improves over time, and early-stage projections of deaths in a pandemic are likely to be unreliable.

The incorporation of behavior data led to an 8% reduction in projection errors during early stage calibration. Mid-stage calibration saw a 79% reduction in errors when behavior data was used, compared to a 36% reduction when it was not used. In late stage calibration, using behavior data led to an 82% reduction in errors, but this reduction was identical to that achieved without behavior data. These findings highlight that the impact of behavior data on projection accuracy is dependent on the availability of sufficient disease data.

In contrast, calibrations using the improper SEIR model exhibit non-monotonic change in projection errors over time. Early stage calibration shows an 11% reduction in errors, but mid stage calibration is 93% higher error than SEIRb’s early calibration. The SEIR model produces better predictions in the early days when data is limited, but its structural deficiencies start affecting projections as time progresses. Late-stage calibration with the SEIR model shows a 36% smaller error, worse than SEIRb’s 82% reduction in error. Despite smaller errors in later stages of the pandemic, the SEIR model’s projections remain less accurate than those of the SEIRb model.

Overall, late-stage SEIRb calibration produced the smallest reduction in projection error (82%) compared to early-stage SEIRb calibration.

### 5.6. Results of sensitivity analysis

Our set of sensitivity tests shows that, overall, the main results are robust in a wide range of conditions (see S1 Text for detailed results). First, the argument that at least one full wave of disease data is required for accurate estimations holds for a wide range of perception delay periods. This is consistently observable by varying delay periods from 20 to 200 days. The main reason is that extending the delay to observe human response also influences the period of first wave of the disease. Second, our three major structural changes also confirm that the behavioral model is still fairly identifiable. The tests included adding seasonality, waning immunity, and variant emergence in the behavioral epidemic model. This is especially helpful given that these sub-structures can create additional oscillations in data, which can overlap the effect of risk-response.

We also test the effects of masking more disease-related parameters in four sets of tests. The results are in line with our main argument that during the early phases, estimating behavioral parameters is prone to error. Having more than one unknown on the disease side of the model makes early estimation of disease parameters also challenging. We extend the tests to different parameter values by changing the values of infectivity rate, sensitivity to death, and time to perceive. Results remain consistent. Finally, we test the effect of having a smaller sample size of contact rate data. The goal was to examine the extent to which behavioral data would be sufficient and useful. Notably, smaller samples of contact rate data from the first wave still provide considerable improvements, showing the significant gain of having any information about public reaction to the disease.

## 6. Discussion and conclusion

We conducted a set of simulation experiments to assess challenges and uncertainties of parameter estimation in behavioral epidemic models with endogenous societal risk-response. Our aim was to examine model identifiability and accurate joint estimation of disease and behavior parameters. Specifically, we hypothesized that the reliability of behavioral parameter estimation is limited before the first wave. Additionally, we investigated the accuracy of parameter estimation under models that overlook behavioral responses. Furthermore, we tested the hypothesis that the addition of data on public behavior, such as observed changes in contact rates in response to the disease, enhances the accuracy of parameter estimates. We projected deaths using the parameter estimates derived from all experiments to examine if the impact is meaningful on projection errors, and to determine which experimental condition yielded the lowest projection error.

Our findings reveal the difficulty in accurately estimating behavioral parameters prior to the first pandemic wave, even in the presence of sufficient disease data and a sound model structure. This challenge arises from the delayed human response to pandemic risks; before the first wave, human reactions are insufficiently triggered, and the data are unaffected by these responses. The societal risk-response takes longer to get triggered compared to other system delays such as the incubation period or disease period [62]. The first waves of the pandemic are mainly driven by changes in risk responses, further complicating the calibration process prior to experiencing the wave. In other words, pre-wave data offers limited insights into people’s response to risks, hindering model calibration. However, a sufficiently extended observation period capturing risk responses enables feasible parameter estimation even in complex systems with additional oscillatory features like seasonality, waning immunity, or variant emergence. This underscores the importance of the timing of societal risk-response delays, setting a minimum data requirement for identifying behavioral parameters. Thus, modelers should exercise patience in projections and explore alternative sources to obtain more direct parameter information.

Furthermore, we find that while the accuracy of predictions improves over time when using an accurate model that better represents reality, this is not the case with a model that improperly neglects change in behavior. In the early days of a disease outbreak, before public reactions have a significant impact on the disease’s state, the SEIR model produces smaller errors than the SEIRb model, despite being an improper model (p<0.001). This is likely due to the fact that the SEIR model has lower degrees of freedom which allows for reasonable estimates of parameter values despite structural deficiencies. This finding suggests that, under certain conditions, an improper model can produce reasonable results. However, the issue is that this can misleadingly create overconfidence for modelers thinking that they can rely on SEIR-like structures that lack behavioral feedback for too long. As time progresses and public behavior changes in response to the disease, the SEIR model’s projection errors increase considerably. The SEIR model cannot generate multiple waves of a pandemic without fine-tuning and changing infectivity rate exogenously. We observe that numerical methods or using larger quantities of data cannot mitigate the impact of ignoring behavioral feedback in models’ structure. As the behavioral feedback in models with endogenous societal risk-response only becomes apparent over time, its exclusion can lead to short-term prediction success but long-term failure at later periods of disease outbreak. These findings emphasize the insufficiency of solely relying on extensive data when models are not properly formulated to incorporate change in human behavior.

While the use of observed data on public behavior has been suggested to enhance the accuracy of pandemic forecasts, our findings indicate that its impact is dependent on the availability of sufficient disease data. Specifically incorporating behavior data in the early stages of a pandemic results in improvements to projection accuracy, but the marginal benefits of having behavioral data decline as the pandemic progresses. These findings emphasize the potential of behavior data, even in limited quantities, for improving early-stage projection accuracy in pandemics.

This study contributes to the growing body of research on behavioral system dynamics [63] and specifically the integration of behavior into dynamic models of infectious diseases [11]. Our analysis, while resonating with several calls for improving validation of behavioral epidemic models [19, 24, 64], shows that the challenges go beyond data availability and include model identifiability limitations.

We recognize several limitations to our approach that provide avenues for further research. Our focus was on a single behavioral epidemic model, and we suggest future studies to analyze parameter estimation in more complex behavioral epidemic models including agent-based structures. Importantly, our study uses a known, true model, whereas in reality, structural uncertainties are greater than what we have assumed, and the set of unknown parameters to be estimated could be larger, posing a greater challenge for accurate parameter estimation. Additionally, inaccuracies in data arising from measurement errors, underreporting or overreporting of cases and deaths were not considered. While we employed the least squares estimation method, using scaled Gaussian and negative binomial methods may provide less biased estimates [49]. However, the choice of estimation method is unlikely to affect the comparison of errors across experimental conditions, which is determined by the model and data rather than the fitting process [50].

Overall, this study points to the challenge of joint estimation of disease and behavioral parameters in a behavioral epidemic model with endogenous societal risk-response. The challenges are related to the observation period, where in early stages it might be infeasible to provide accurate estimation of the parameter values, and to the use of improper models for model calibration. While gathering extensive and accurate data is required, it is not sufficient for providing accurate projections.

## Supporting information

S1 Text Additional model details and results. S1 Text comprises ten sections: 1) model equations; 2) list of parameter values used to generate synthetic data; 3) parameter estimation method used in experiments; 4) performance metrics for deaths projection; 5) statistical test results for main experiments; 6) sensitivity analysis to length of delay in human response to risk; 7) sensitivity analysis to other oscillatory phenomena; 8) sensitivity analysis for other disease parameters; 9) sensitivity analysis to different parameter values, and 10) sensitivity analysis to amount of contact rate data.
(DOCX)
