# Supplementary material for: Parameter estimation in behavioral epidemic models with endogenous societal risk-response
Source: PLoS Comput Biol. 2024 Mar 29;20(3):e1011992. doi: 10.1371/journal.pcbi.1011992 (PMC11006122; doi:10.1371/journal.pcbi.1011992)
Supplement: S1 Text — comprises ten sections: 1) model equations; 2) list of parameter values used to generate synthetic data; 3) parameter estimation method used in experiments; 4) performance metrics for deaths projection; 5) statistical test results for main experiments; 6) sensitivity analysis to length of delay in human response to risk; 7) sensitivity analysis to other oscillatory phenomena; 8) sensitivity analysis for other disease parameters; 9) sensitivity analysis to different parameter values, and 10) sensitivity analysis to amount of contact rate data. (DOCX) [file pcbi.1011992.s001.docx]

S1 Text.

Supplementary Information for:

**Parameter estimation in behavioral epidemic models with endogenous societal risk-response**

Ann Osi, Navid Ghaffarzadegan

Table of Contents

[1: Model Equations 3](#_Toc155778640)

[2: True Parameter Values 4](#_Toc155778641)

[3: Parameter Estimation Method 5](#_Toc155778642)

[4: Deaths Prediction 7](#_Toc155778643)

[5: Statistical Tests of Main Experiments 8](#_Toc155778644)

[6: Sensitivity analysis to length of delay in human response to risk 10](#_Toc155778645)

[7: Sensitivity analysis to other oscillatory phenomena 11](#_Toc155778646)

[8: Sensitivity analysis for other disease parameters. 15](#_Toc155778647)

[9: Sensitivity analysis to different parameter values. 16](#_Toc155778648)

[10: Sensitivity analysis to amount of contact rate data. 17](#_Toc155778649)

[References 19](#_Toc155778650)

## 1: Model Equations

As stated, the core structure of the model comes from previously validated behavioral epidemic model [1]. The model was formulated in Vensim DSS Software. Its equations can be used to rebuild the model in other software or applications. We provide the model in Vensim for readers familiar with the software. Refer to <https://zenodo.org/records/10501198>

Equations 1-4 depict the main structure of the model, where N is population, S is susceptible, E is exposed, I is infected, and R is removed.

| $\frac{dS}{dt}=- \beta\frac{SI}{N}\theta_{n}$ | (1) |
| --- | --- |
| $\frac{dE}{dt}=\beta\frac{SI}{N}\theta_{n}-\frac{E}{\lambda_{e}}$ | (2) |
| $\frac{dI}{dt}=\frac{E}{\lambda_{e}}-\frac{I}{\lambda_{i}}$ | (3) |
| $\frac{dR}{dt}=\frac{I}{\lambda_{i}}$ | (4) |

where $\beta$ is the infectivity rate, which is the rate at which the infected population causes new infections, $\lambda_{e}$ is the average exposure period until symptom onset, $\lambda_{i}$ is the average infection period from symptom onset to death or recovery, and $\theta_{n}$ is the correlated noise distribution.

The risk responsiveness of the public to change in the state of the pandemic is represented by a feedback loop [2-4]. Specifically, the infectivity rate, $\beta$, varies with time as individuals alter their interactions in response to changes in their perceived risk of death. Specifically, the perceived risk of death $\left( f^{'} \right)$ is modeled as a delayed function of daily deaths $\left( f \right)$, and the degree to which individuals adjust contacts, $k$, is dependent on the population’s sensitivity to changes in perceived risk. This is mathematically expressed in equations 5-8.

| $f=r\frac{dR}{dt}$ | (5) |
| --- | --- |
| $\frac{df^{'}}{dt}=\frac{f^{'}-f}{\lambda_{p}}$ | (6) |
| $k= EXP\left( -\alpha f^{'} \right)$ | (7) |
| $\beta={k\beta}_{0}$ | (8) |

where $r$ is the infected fatality rate, which is the probability of death after infection, $\lambda_{p}$, referred to as time to perceive, measures the delay in behavior adaptation in response to risk perception, and $\alpha$, referred to as sensitivity to risk, measures the degree of response to risk perception.

## 2: True Parameter Values

Table S1.1 reports the parameter values used to create synthetic data to help replication of our findings. The parameter values are selected within realistic ranges for COVID-19 pandemic, for example the incubation and infection periods are set at 3 and 10 days, and the initial reproductive number is about 4 [5].

Table S1.1: Parameter values used to create synthetic data.

| Parameter | Unit | Value |
| --- | --- | --- |
| Initial population $\left( \boldsymbol{N}\left( \boldsymbol{0} \right) \right)$ | Person | 1,000,000 |
| Initial Susceptible $\left( \boldsymbol{S}\left( \boldsymbol{0} \right) \right)$ | Person | 999,999 |
| Initial Exposed $\left( \boldsymbol{E}\left( \boldsymbol{0} \right) \right)$ | Person | 1 |
| Initial Infected $\left( \boldsymbol{I}\left( \boldsymbol{0} \right) \right)$ | Person | 0 |
| Initial Recovered $\left( \boldsymbol{R}\left( \boldsymbol{0} \right) \right)$ | Person | 0 |
| Noise correlation time $\left( \boldsymbol{s}_{\boldsymbol{C}} \right)$ | Day | 15 |
| Noise standard deviation $\left( \boldsymbol{s}_{\boldsymbol{d}} \right)$ | Dmnl | 0.3 |
| Noise mean $\left( \boldsymbol{s}_{\boldsymbol{m}} \right)$ | Dmnl | 1 |
| Exposure period ($\boldsymbol{\lambda}_{\boldsymbol{e}}$) | Day | 3 |
| Infection period ($\boldsymbol{\lambda}_{\boldsymbol{i}}\boldsymbol{)}$ | Day | 10 |
| Initial infectivity rate $\left( \boldsymbol{\beta}_{\boldsymbol{o}} \right)$ | Dmnl | 0.4 |
| Infected fatality rate $\left( \boldsymbol{r} \right)$ | Dmnl | 0.005 |
| Sensitivity to death $\left( \boldsymbol{\alpha} \right)$ | Dmnl | 0.5 |
| Time to perceive ($\boldsymbol{\lambda}_{\boldsymbol{p}}\boldsymbol{)}$ | Day | 60 |

Dmnl = dimensionless.

## 3: Parameter Estimation Method

Parameter estimation is implemented using Vensim DSS’s optimization function, which employs a Powell hill-climbing algorithm to search over a range of parameter values and find the value that minimizes the weighted sum of squared errors between model output and data, as described in equation 9. This method assumes that the errors are independent, identically distributed normal random variables with constant variance. It is a widely used technique for parameter estimation in this domain [1, 6].

| ${MIN}_{p}\left( \sum_{ijt} w_{ij}\left( y_{ijt}-\hat{y}_{ijt} \right)^{2} \right)$ | (9) |
| --- | --- |

where $y_{ijt}$ = value of dataset (i)-stochastic realization(j) pair at time t, $\hat{y}_{ijt}$ = simulation values for the value of dataset (i)-stochastic realization(j) pair at time t, $p$ = model parameters to be estimated, $w_{ij}$ = weights associated with each dataset-stochastic realization pair (calculated as 1/standard deviation).

The method is conducted as follows:

1. **Payoff function definition:**

- We specified model variables, data, and weights for the optimization function in equation 9 inside a Vensim Payoff Definition (*.vpd*) file.
- For experiments with only one data variable, the weight is 1 for all stochastic data realizations. For experiments with multiple data variables, the weight is calculated as 1/standard deviation for each dataset-stochastic realization pair.

1. **Optimization options:**
   - We specified parameters for optimization, search ranges for each parameter, and initial starting points for the search inside a Vensim Optimization Control (*.voc*) file.
   - The same starting values (assumed distant from ground truth) and search ranges were employed across all calibrations.
   - To prevent sensitivity to starting values, the optimizer was configured to initiate new optimizations randomly and uniformly within each parameter's range.
   - Table S1.2 outlines the specific optimization options used.
   - Table S1.3 provides comprehensive details regarding starting values and search ranges.

**Replication guidance:**

- - For Vensim users: Payoff definition and optimization control files are available at <https://zenodo.org/records/10501198>
  - For other software/applications: The information in Tables A1- A3 enable replication within alternative environments.
  - For further information: Consult the free Vensim user guide.

Table S1.2: Optimization options

| OPTIMIZER=Powell | MCBURNIN=0 |
| --- | --- |
| SENSITIVITY=Off | MCDELTA=0.0001 |
| MULTIPLE_START=Random | MCTEMP=1 |
| RANDOM_NUMBER=Default | MCNCHAINS=2 |
| OUTPUT_LEVEL=On | MCUPDATEPAIRS=2 |
| TRACE=Off | MCSCHEDULE=0 |
| MAX_ITERATIONS=1000 | MCOUTLIER=0.05 |
| RESTART_MAX=10 | MCXOVER=0.2 |
| PASS_LIMIT=2 | MCCOOLING=1000 |
| FRACTIONAL_TOLERANCE=0.0003 | MCINITMETHOD=0 |
| TOLERANCE_MULTIPLIER=21 | MCPAYOFFTYPE=0 |
| ABSOLUTE_TOLERANCE=1 | MCGAMMA=1 |
| SCALE_ABSOLUTE=1 | MCRECORD=0 |
| VECTOR_POINTS=25 | MCKNN=0 |
| MCLIMIT=0 | MCEPSILON=0.01 |
| MCJUMP=0.05 | MCFTEMP=1 |

Table S1.3: Initial values and ranges for parameter estimation

| Parameters | Initial Value | Minimum Value | Maximum Value |
| --- | --- | --- | --- |
| Infectivity rate (Beta) (β) | 0.8 | 0 | 1.5 |
| Sensitivity to death (*α*) | 1.0 | 0 | 1.5 |
| Time to perceive ($\boldsymbol{\lambda}_{\boldsymbol{p}}\boldsymbol{)}$ | 35 | 0 | 100 |

## 4: Deaths Prediction

The prediction error (PE) is calculated as the daily mean absolute percentage error between simulated deaths ($\hat{y}_{t}$) and actual deaths ($y_{t}$) cumulated over 365 days (equation 10).

| $PE=\sum_{t=t_{f}+1}^{t_{f}+u} s^{-1}\sum_{j=1}^{s} \left\vert\frac{\hat{y}_{jt}-y_{jt}}{y_{jt}} \right\vert$ | (10) |
| --- | --- |

where $u$= prediction interval (365 days), $t_{f}$ = final simulation time, $s$= number of stochastic data series (100), $\hat{y}_{jt}$ = model output j at time t, $y_{jt}$= data for model output j at time t.

## 5: Statistical Tests of Main Experiments

We show the results of paired t-tests comparing average absolute parameter estimation errors between groups of different experiments in Tables A4-A6. The average absolute parameter estimation error is the average absolute difference between the estimated and true parameter values. The group difference represents the difference in the average absolute parameter estimation error between group 1 and group 2. A positive group difference value indicates that the error of group 1 is higher.

Table S1.4: Paired t-tests comparing observation time pairs of the SEIRb model.

| Parameter | Group 1 | Group 2 | Group difference | 95% Confidence Interval | | p-value |
| --- | --- | --- | --- | --- | --- | --- |
| Infectivity rate | Early-60 days | Mid-120 days | 0.001 | -0.004 | 0.006 | 0.634 |
| Infectivity rate | Early-60 days | Late-365 days | 0.004 | -0.002 | 0.010 | 0.203 |
| Infectivity rate | Mid-120 days | Late-365 days | 0.003 | 0.001 | 0.005 | 0.011* |
| Sensitivity to death | Early-60 days | Mid-120 days | 0.283 | 0.223 | 0.340 | <0.001*** |
| Sensitivity to death | Early-60 days | Late-365 days | 0.383 | 0.333 | 0.433 | <0.001*** |
| Sensitivity to death | Mid-120 days | Late-365 days | 0.100 | 0.075 | 0.125 | <0.001*** |
| Time to perceive | Early-60 days | Mid-120 days | 6.571 | 2.786 | 10.356 | <0.001*** |
| Time to perceive | Early-60 days | Late-365 days | 15.802 | 11.702 | 19.903 | <0.001*** |
| Time to perceive | Mid-120 days | Late-365 days | 9.232 | 6.007 | 12.456 | <0.001*** |

***p<0.001, ** p<0.01, * p<0.05

Table S1.5: Paired t-tests between the SEIRb and SEIR models at different observation time.

| Parameter | Observation time | Group 1 | Group 2 | Group difference | 95% Confidence Interval | | p-value |
| --- | --- | --- | --- | --- | --- | --- | --- |
| Infectivity rate | Early-60 days | SEIRb | SEIR | 0.009 | 0.004 | 0.014 | <0.001*** |
| Infectivity rate | Mid-120 days | SEIRb | SEIR | -0.028 | -0.035 | -0.021 | <0.001*** |
| Infectivity rate | Late-365 days | SEIRb | SEIR | -0.206 | -0.214 | -0.198 | <0.001*** |

***p<0.001, ** p<0.01, * p<0.05

Table S1.6: Paired t-tests for the SEIRb model with and without using data on public behavior for calibration.

| Parameter | Observation Period | Group 1 | Group 2 | Group difference | 95% CI | | p-value |
| --- | --- | --- | --- | --- | --- | --- | --- |
| Infectivity rate | Early-60 days | SEIRb | SEIRb-with contact rate | 0 | -0.0025 | 0.003 | 0.8151 |
| Infectivity rate | Mid-120 days | SEIRb | SEIRb-with contact rate | 0.037 | 0.001 | 0.006 | 0.002** |
| Infectivity rate | Late-365 days | SEIRb | SEIRb-with contact rate | 0.002 | 0 | 0.003 | 0.008** |
| Sensitivity to death | Early-60 days | SEIRb | SEIRb-with contact rate | 0.285 | 0.231 | 0.338 | <0.001*** |
| Sensitivity to death | Mid-120 days | SEIRb | SEIRb-with contact rate | 0.062 | 0.029 | 0.096 | <0.001*** |
| Sensitivity to death | Late-365 days | SEIRb | SEIRb-with contact rate | 0 | -0.004 | 0.005 | 0.9078 |
| Time to perceive | Early-60 days | SEIRb | SEIRb-with contact rate | 4.294 | 0.573 | 8.015 | 0.024* |
| Time to perceive | Mid-120 days | SEIRb | SEIRb-with contact rate | -8.144 | -11.748 | -4.541 | <0.001*** |
| Time to perceive | Late-365 days | SEIRb | SEIRb-with contact rate | -0.054 | -0.388 | 0.281 | 0.751 |

*** p<0.001, ** p<0.01, * p<0.05

## 6: Sensitivity analysis to length of delay in human response to risk

We perform an additional experiment to test if the results change for different lengths of delay in human response to risk. Ten datasets are generated by employing a unique noise seed for each and varying the time to perceive value, $\boldsymbol{\lambda}_{\boldsymbol{p}}$, ranging from $\boldsymbol{\lambda}_{\boldsymbol{p}}=20$ to $\boldsymbol{\lambda}_{\boldsymbol{p}}=200$ in 20-day increments of 20 (i.e., 20, 40, 60, ..., 200). Calibration is then performed at three different time periods: t, which corresponds to the onset of the delay period (e.g., If $\lambda_{p}=20,$then $t=20$); time $2t$ which represents double the delay period; and time $6t$, corresponding to six times the delay period. Estimation errors are calculated as the absolute percentage error between the estimated parameter values and the true parameter values.

The distribution of the estimation errors for each period is illustrated in Figure S1.1. Figure S1.1 shows that the estimation error for the infectivity rate (β_o_) remains consistent across all time periods. In contrast, the estimation error for behavior parameters decreases as time progresses from the delay period (i.e. after onset). This indicates that even with extensive, accurate data, estimation of behavior parameters hinges on the timing of the delay in behavioral response, thus supporting H1b.


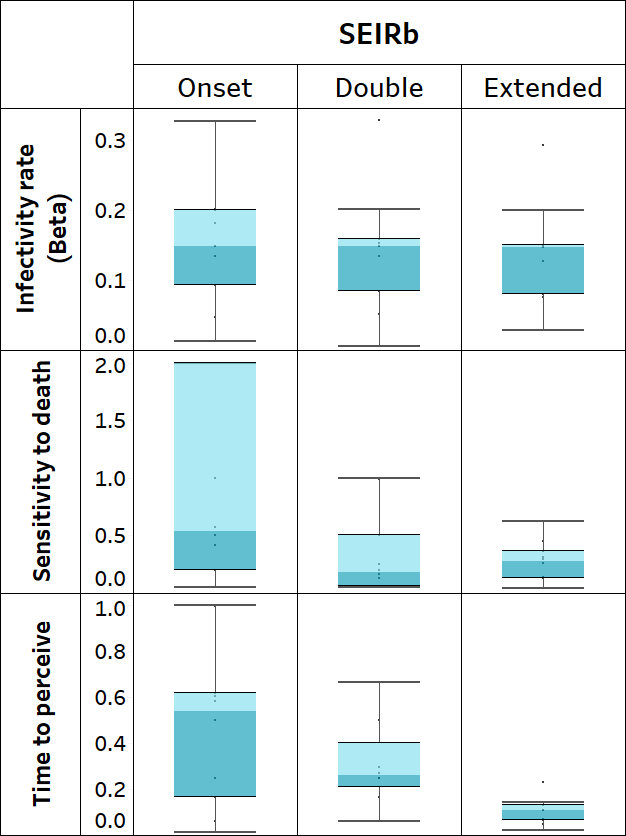


Figure S1.1. Distribution of parameter estimation errors from calibrating the SEIRb model using data created with varying delay in risk perception (20-200 days) and three observation period (Onset: t, Double: 2t, Extended: 6t).

## 7: Sensitivity analysis to other oscillatory phenomena

Disease dynamics are influenced not only by behavioral changes but also by seasonality [7], , loss of immunity [8], and emergence of variants [9]. These features introduce new challenges in identifying parameters as data oscillations are influenced by both behavior and these additional features. Additionally, modelers need to expand model equations and variables to incorporate these new dynamics, resulting in an increased number of parameters to be jointly estimated. To test the effects of these additional dynamics, we enhance the SEIRb model by incorporating seasonality, loss of immunity, and emergence of variants individually.

***Seasonality:***

The model equations (equations 1-7) remain the same. However, the infectivity rate, $\beta$, is modified to be a function of seasonality (equations 11-12) as done in [10].

| $\phi=1+\beta_{1}cos\left( \frac{2\pi t}{365} \right)$ | (11) |
| --- | --- |
| $\beta={\phi k\beta}_{0}$ | (12) |

where ϕ is the seasonality effect, and β_1 is the amplitude of seasonal forcing.

Seasonal variations differ greatly across regions. To explore this variability, we conduct two experiments: one with a moderate amplitude of 0.3 and another with a pronounced amplitude of 0.7. The amplitude is assumed unknown and included in the set of estimated parameters.

***Loss of immunity:***

The model equations (equations 1-4) are adjusted as shown by equations 13-16 to include waning immunity after recovery, $\omega$. Waning immunity is modeled as a third-order exponential delay of recovery, $r$, occurring over a waning period, $\lambda_{\omega}$. During this period, individuals gradually lose immunity and regain susceptibility to infection (equations 17-20).

| $\frac{dS}{dt}=\omega- \beta\frac{SI}{N}\theta_{n}$ | (13) |
| --- | --- |
| $\frac{dE}{dt}=\beta\frac{SI}{N}\theta_{n}-\frac{E}{\lambda_{e}}$ | (14) |
| $\frac{dI}{dt}=\frac{E}{\lambda_{e}}-\frac{I}{\lambda_{i}}$ | (15) |
| $\frac{dR}{dt}=\frac{I}{\lambda_{i}}-\omega$ | (16) |
| $r=(1-f)\frac{I}{\lambda_{i}}$ | (17) |
| $\frac{{d\omega,}_{1}}{dt}=r-\frac{\omega_{,1}}{\frac{\lambda_{\omega}}{3}}$ | (18) |
| $\frac{{d\omega}_{, 2}}{dt}=\frac{{\omega,}_{1}}{\frac{\lambda_{\omega}}{3}}-\frac{\omega_{,2}}{\frac{\lambda_{\omega}}{3}}$ | (19) |
| $\frac{{d\omega,}_{3}}{dt}=\frac{\omega_{,3}}{\frac{\lambda_{\omega}}{3}}-\frac{\omega_{,2}}{\frac{\lambda_{\omega}}{3}}$ | (20) |

We conduct an experiment with a waning period of 280 days similar to estimates for COVID-19 [11]. The waning period is assumed unknown and included in the set of estimated parameters.

***Variant Emergence:***

The model equations (equations 1-7) remain the same. However, the initial infectivity rate, $\beta_{0}$, rises linearly by a factor, $\Delta\beta$, over 60 days upon variant emergence and remains constant afterwards (equations 21-23).

|  |  |
| --- | --- |
| $s=\left\{ \begin{aligned} 0, for t\leq\lambda_{\nu} \\ \frac{t-\lambda_{\nu}}{60}, for \lambda_{\nu}<t\leq\lambda_{\nu}+60 \\ 1, for t>\lambda_{\nu}+60 \end{aligned} \right.$ | (21) |
| $\nu=s\beta_{0}(\Delta\beta-1)$ | (22) |
| $\beta={k(\beta}_{0}+\nu)$ | (23) |

where $s$ is the slope of change in infectivity rate during variant emergence, $\lambda_{\nu}$ is the variant emergence time, and $\nu$ is the change in infectivity rate during variant emergence.

We simulate the emergence of a COVID-19 Delta-like variant [12], where infectivity increases by 150% over 60 days, starting on day 150. The change in infectivity is assumed unknown and included in the set of estimated parameters.

We compare all four experiments to the base case with only behavioral changes (from section 5.1). All experiments used 365-day datasets to assess whether behavioral parameters could be reliably estimated when additional oscillatory dynamics are present. Figure S1.2 shows efficient estimation for all parameters except the SEIRb model with high seasonality. Table S1.7 reveals significant differences between the base case and the high seasonality experiment. Therefore, with very high amplitudes, 365 days may not be sufficient for reliable parameter estimation and a longer time series might be needed.


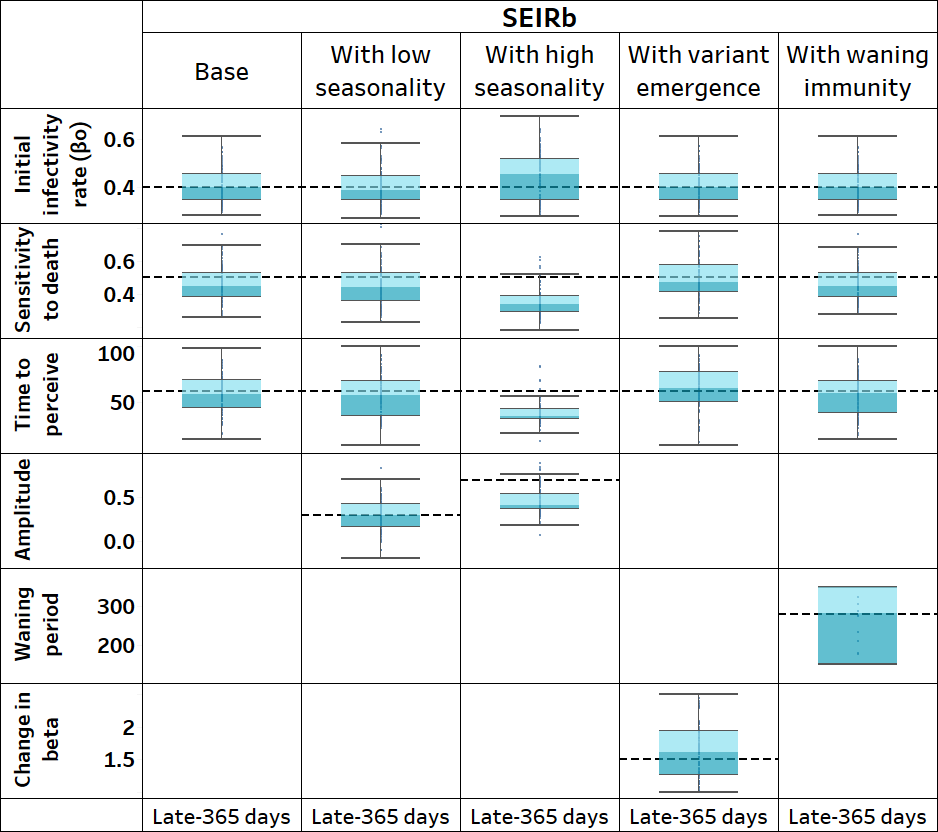


Figure S1.2. Estimated parameter values for sensitivity analysis to other oscillatory phenomena. The Base model is the SEIRb model. Other models are SEIRb models with added oscillatory dynamics. Actual parameter values are indicated by broken lines.

The average absolute parameter estimation error is the average absolute difference between the estimated and true parameter values. The group difference represents the difference in the average absolute parameter estimation error between group 1 and group 2. A positive group difference value indicates that the error of group 1 is higher.

Table S1.7: T-tests comparing SEIRb model to SEIRb models with additional oscillatory dynamics.

| Parameter | Group 1 | Group 2 | 95% Confidence Interval | | p-value |
| --- | --- | --- | --- | --- | --- |
| Infectivity rate | Base | With low seasonality | -0.018 | 0.009 | 0.530 |
| Infectivity rate | Base | With high seasonality | -0.047 | -0.019 | <0.001*** |
| Infectivity rate | Base | With loss of immunity | -0.011 | 0.011 | 0.998 |
| Infectivity rate | Base | With variant emergence | -0.013 | 0.010 | 0.835 |
| Sensitivity to death | Base | With low seasonality | -0.037 | 0.002 | 0.086 |
| Sensitivity to death | Base | With high seasonality | -0.084 | -0.047 | <0.001*** |
| Sensitivity to death | Base | With loss of immunity | -0.014 | 0.021 | 0.709 |
| Sensitivity to death | Base | With variant emergence | -0.021 | 0.015 | 0.773 |
| Time to perceive | Base | With low seasonality | -5.270 | 0.656 | 0.126 |
| Time to perceive | Base | With high seasonality | -9.855 | -4.947 | <0.001*** |
| Time to perceive | Base | With loss of immunity | -3.832 | 1.910 | 0.509 |
| Time to perceive | Base | With variant emergence | -6.538 | -0.092 | 0.044* |

***p<0.001, ** p<0.01, * p<0.05. The p-value is the probability that the average estimation error of the two groups is statistically the same. Average estimation errors using the SEIRb model with high seasonality is significantly different from the SEIRb model with only behavior for all parameter values.

## 8: Sensitivity analysis for other disease parameters.

We conducted four experiments with unknown incubation and infection periods and paired with an unknown infectivity rate. These were compared to the base case with only the infectivity rate unknown (Section 5.1). We evaluated parameter estimation early in the pandemic.

Figure S1.3 shows efficient estimation when only one disease parameter is unknown, and unreliable estimation when two disease parameters are unknown. Results also align with previous findings that behavioral parameters are unreliable with 60 days of data.


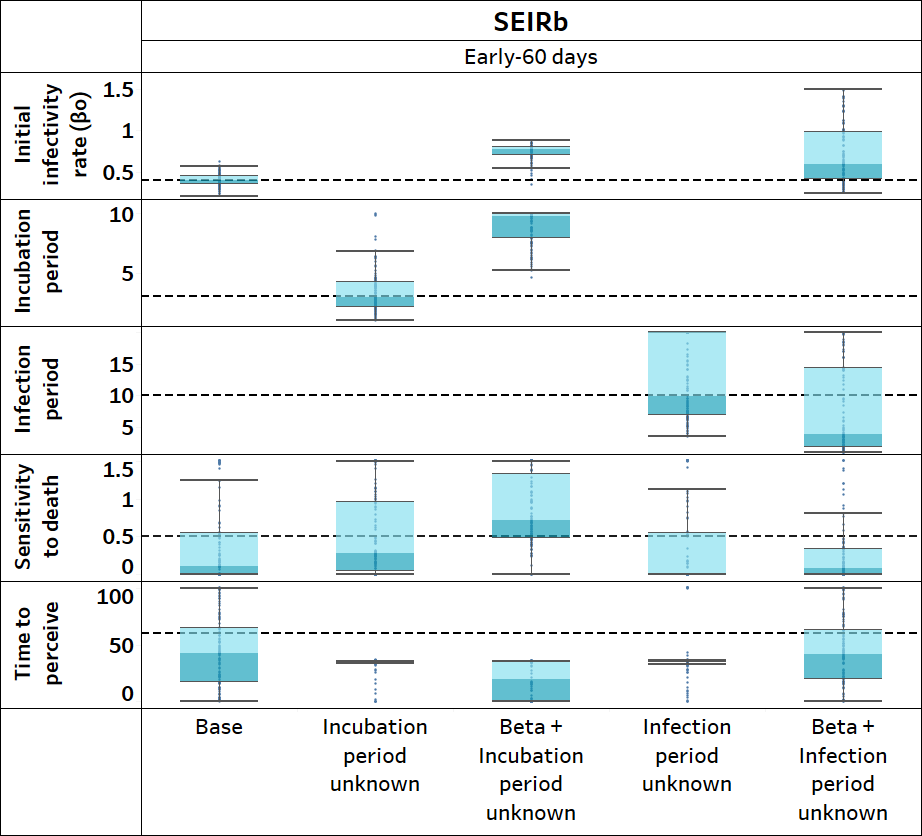


Figure S1.3. Estimated parameter values for sensitivity analysis when disease parameters are unknown. In the Base case, infectivity rate is the only unknown disease parameter. Actual parameter values are indicated by broken lines.

## 9: Sensitivity analysis to different parameter values.

To assess generalizability of results in section 5.1, we conducted additional experiments varying true parameter values: infectivity rate (+/- 50%), sensitivity to death (+/- 60%), and time to perceive (+/- 50%). Figure S1.4 shows that disease parameters are consistently estimated efficiently, while behavioral parameters remain challenging to estimate early in the pandemic, regardless of specific parameter values.


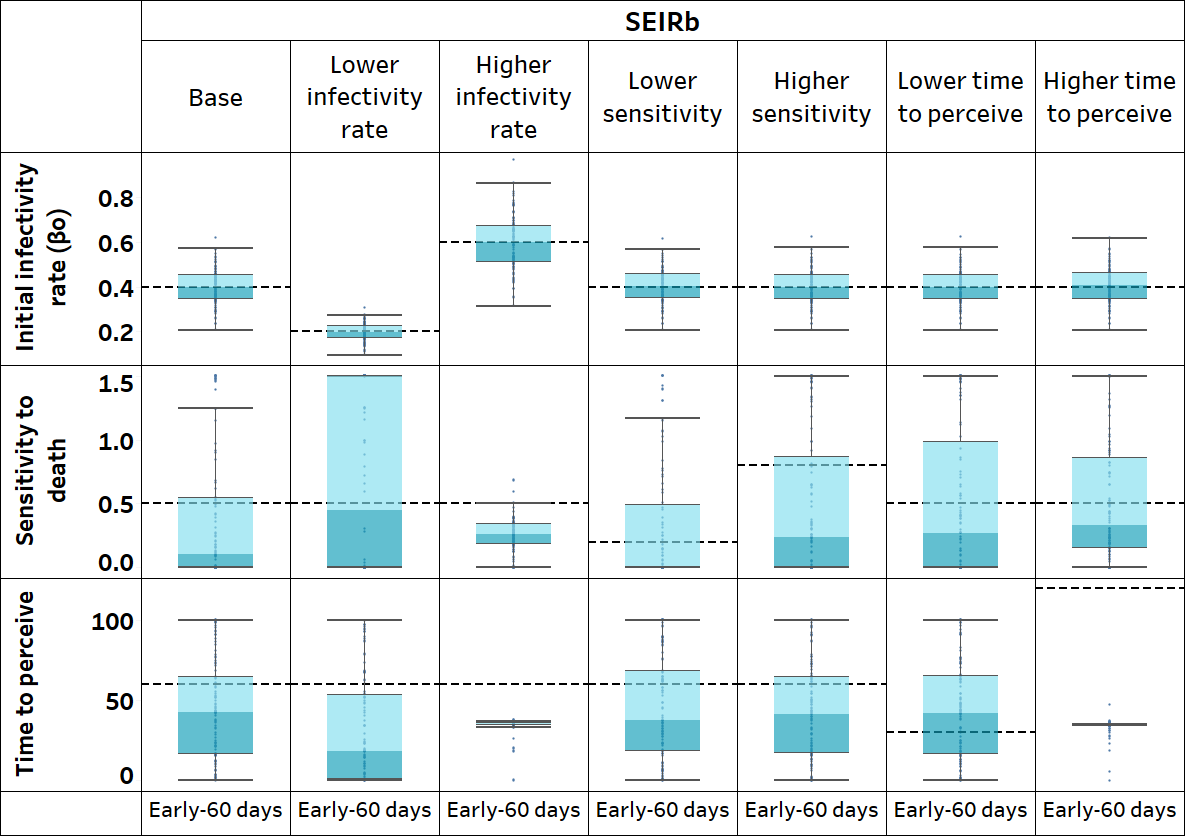


Figure S1.4. Estimated parameter values for sensitivity analysis to different parameter values. Actual parameter values are indicated by broken lines.

## 10: Sensitivity analysis to amount of contact rate data.

To assess the importance of contact rate data, we used two shorter time series: one from the exponential death rise in the first wave (days 30-60) and another even shorter (days 51-57). Figure S1.5 shows that parameter estimates with these limited datasets closely mirror those from Section 5.3, where contact rate data was available for the entire observation period. Notably, Table S1.7 reveals no significant differences in many estimates between the two scenarios and the main experiment results. Furthermore, the overall trends remain consistent with Section 5.3.


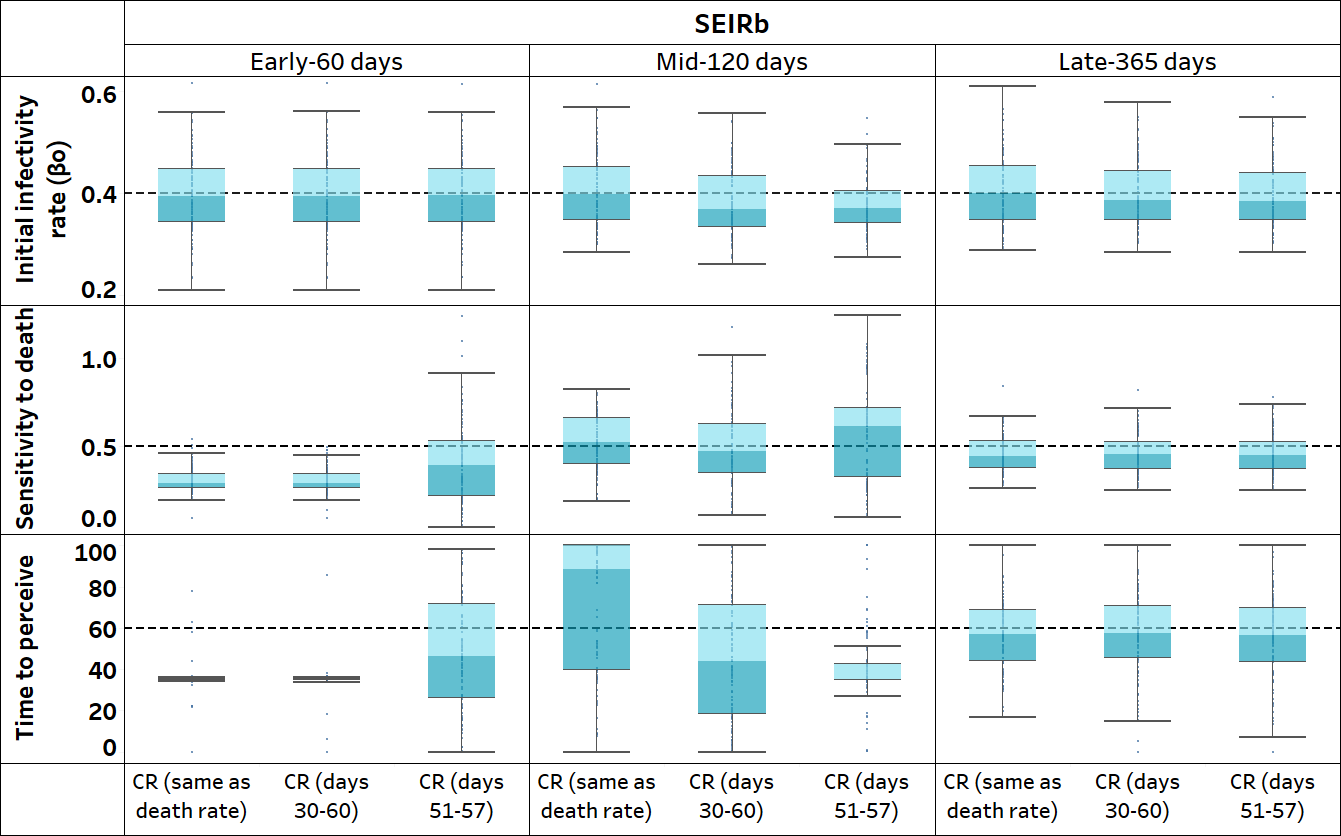
Figure S1.5. Estimated parameter values for sensitivity analysis to amount of contact rate data (CR). Actual parameter values are indicated by broken lines.

Table S1.7: Paired t-tests for the SEIRb model with different amount of contact rate data

| Parameter | Observation Period | Group 1 | Group 2 | Group Difference | 95% Confidence Interval | | p-value |
| --- | --- | --- | --- | --- | --- | --- | --- |
| Infectivity rate | Early-60 days | Days 1-60 | Days 30-60 | 0.000 | 0.000 | 0.000 | 0.429 |
| Infectivity rate | Mid-120 days | Days 1-120 | Days 30-60 | -0.002 | -0.007 | 0.004 | 0.521 |
| Infectivity rate | Late-365 days | Days 1-365 | Days 30-60 | 0.032 | 0.000 | 0.006 | 0.04* |
| Infectivity rate | Early-60 days | Days 1-60 | Days 51-57 | 0.000 | 0.000 | 0.000 | 0.006** |
| Infectivity rate | Mid-120 days | Days 1-120 | Days 51-57 | 0.011 | 0.005 | 0.018 | <0.001*** |
| Infectivity rate | Late-365 days | Days 1-365 | Days 51-57 | 0.004 | 0.001 | 0.008 | 0.015* |
| Sensitivity to death | Early-60 days | Days 1-60 | Days 30-60 | -0.003 | -0.012 | 0.006 | 0.499 |
| Sensitivity to death | Mid-120 days | Days 1-120 | Days 30-60 | -0.064 | -0.103 | -0.025 | 0.001* |
| Sensitivity to death | Late-365 days | Days 1-365 | Days 30-60 | -0.002 | -0.012 | 0.008 | 0.633 |
| Sensitivity to death | Early-60 days | Days 1-60 | Days 51-57 | -0.013 | -0.047 | 0.021 | 0.463 |
| Sensitivity to death | Mid-120 days | Days 1-120 | Days 51-57 | -0.110 | -0.147 | -0.733 | <0.001*** |
| Sensitivity to death | Late-365 days | Days 1-365 | Days 51-57 | -0.006 | -0.017 | 0.005 | 0.261 |
| Time to perceive | Early-60 days | Days 1-60 | Days 30-60 | -0.656 | -1.747 | 0.435 | 0.236 |
| Time to perceive | Mid-120 days | Days 1-120 | Days 30-60 | 3.051 | -1.103 | 7.205 | 0.148 |
| Time to perceive | Late-365 days | Days 1-365 | Days 30-60 | -2.164 | -3.927 | -0.401 | 0.017* |
| Time to perceive | Early-60 days | Days 1-60 | Days 51-57 | -0.045 | -3.593 | 3.503 | 0.98 |
| Time to perceive | Mid-120 days | Days 1-120 | Days 51-57 | 6.966 | 3.548 | 10.385 | <0.001*** |
| Time to perceive | Late-365 days | Days 1-365 | Days 51-57 | -2.312 | -4.070 | -0.553 | 0.010** |

***p<0.001, ** p<0.01, * p<0.05. The p-value is the probability that the average estimation error of pairs of the two groups is statistically the same. Average estimation errors when contact rate data is available for the entire observation period are not significantly different from errors when limited contact rate data is used for majority of the experiments.

# References

1. Rahmandad H, Xu R, Ghaffarzadegan N. Enhancing long-term forecasting: Learning from COVID-19 models. PLoS Comput Biol. 2022;18(5):e1010100. Epub 20220519. doi: 10.1371/journal.pcbi.1010100. PubMed PMID: 35587466; PubMed Central PMCID: PMCPMC9119494.

2. Ferguson N. Capturing human behaviour. Nature. 2007;446(7137):733-.

3. Funk S, Salathé M, Jansen VA. Modelling the influence of human behaviour on the spread of infectious diseases: a review. Journal of the Royal Society Interface. 2010;7(50):1247-56.

4. Rahmandad H, Xu R, Ghaffarzadegan N. A missing behavioural feedback in COVID-19 models is the key to several puzzles. BMJ Global Health. 2022;7(10):e010463.

5. COVID-19 Pandemic Planning Scenarios Centers for Disease Control and Prevention2021. Available from: <https://www.cdc.gov/coronavirus/2019-ncov/hcp/planning-scenarios.html>.

6. Capaldi A, Behrend S, Berman B, Smith J, Wright J, Lloyd AL. Parameter estimation and uncertainty quantication for an epidemic model. Mathematical biosciences and engineering. 2012:553.

7. Xu R, Rahmandad H, Gupta M, DiGennaro C, Ghaffarzadegan N, Amini H, et al. Weather, air pollution, and SARS-CoV-2 transmission: a global analysis. The Lancet Planetary Health. 2021;5(10):e671-e80.

8. Hall V, Foulkes S, Insalata F, Kirwan P, Saei A, Atti A, et al. Protection against SARS-CoV-2 after Covid-19 vaccination and previous infection. New England Journal of Medicine. 2022;386(13):1207-20.

9. Rahmandad H, Sterman J. Quantifying the COVID‐19 endgame: Is a new normal within reach? System Dynamics Review. 2022;38(4):329-53.

10. Liu X, Huang J, Li C, Zhao Y, Wang D, Huang Z, et al. The role of seasonality in the spread of COVID-19 pandemic. Environmental research. 2021;195:110874.

11. Stein C, Nassereldine H, Sorensen RJ, Amlag JO, Bisignano C, Byrne S, et al. Past SARS-CoV-2 infection protection against re-infection: a systematic review and meta-analysis. The Lancet. 2023;401(10379):833-42.

12. Emergencies SAGf. SPI-MO: consensus statement on COVID-19, 3 June 2021. 2021.
